# Author Correction: Genetic mapping of cell type specificity for complex traits

**DOI:** 10.1038/s41467-020-15365-y

**Published:** 2020-04-01

**Authors:** Kyoko Watanabe, Maša Umićević Mirkov, Christiaan A. de Leeuw, Martijn P. van den Heuvel, Danielle Posthuma

**Affiliations:** 1 Department of Complex Trait Genetics, Center for Neurogenomics and Cognitive Research, Neuroscience Campus Amsterdam, VU University Amsterdam, Amsterdam, The Netherlands; 2 Department of Clinical Genetics, section Complex Trait Genetics, Neuroscience Campus Amsterdam, VU Medical Center, Amsterdam, The Netherlands

**Keywords:** Computational biology and bioinformatics, Gene expression, Genome-wide association studies

Correction to: *Nature Communications* 10.1038/s41467-019-11181-1, published online 19 July 2019.

The original version of this Article contained an error in Fig. 5 and Supplementary Fig. 12, in which the colours of the bar plots at the top of the heatmaps were incorrectly given, leading to incorrect mapping of the plots to the corresponding dataset based on the dataset legend.

The correct version of Fig. 5 is:  which replaces the previous incorrect version:This has been corrected in both the PDF and HTML versions of the Article.

The correct version of Supplementary Fig. 12 is: which replaces the previous incorrect version:. The HTML has been updated to include a corrected version of the Supplementary Information.

