# Supplementary material for: Genetic mapping of cell type specificity for complex traits
Source: Nat Commun. 2019 Jul 19;10:3222. doi: 10.1038/s41467-019-11181-1 (PMC6642112; doi:10.1038/s41467-019-11181-1)
Supplement: Supplementary file 1 — Supplementary Information [file 41467_2019_11181_MOESM1_ESM.pdf]

Supplementary Information for:

Genetic mapping of cell type specificity for complex traits

Watanabe et al.

Table of contents

Supplementary Figures 3

Supplementary Tables 24

Supplementary Table 1. Threshold of P-value and proportional significance for conditional analysis 24

Supplementary Table 2. GWAS summary statistics for 26 traits 25

Supplementary Note 27

1. Effects of average expression across cell types 27

2. Comparison with the model from the study of Skene *et al.* 27

3. Comparison with stratified LD score regression and RolyPoly 30

4. FDR versus Bonferroni multiple testing correction 31

5. Detailed interpretation of multi-conditional analyses 31

Supplementary References 34

Supplementary Figures

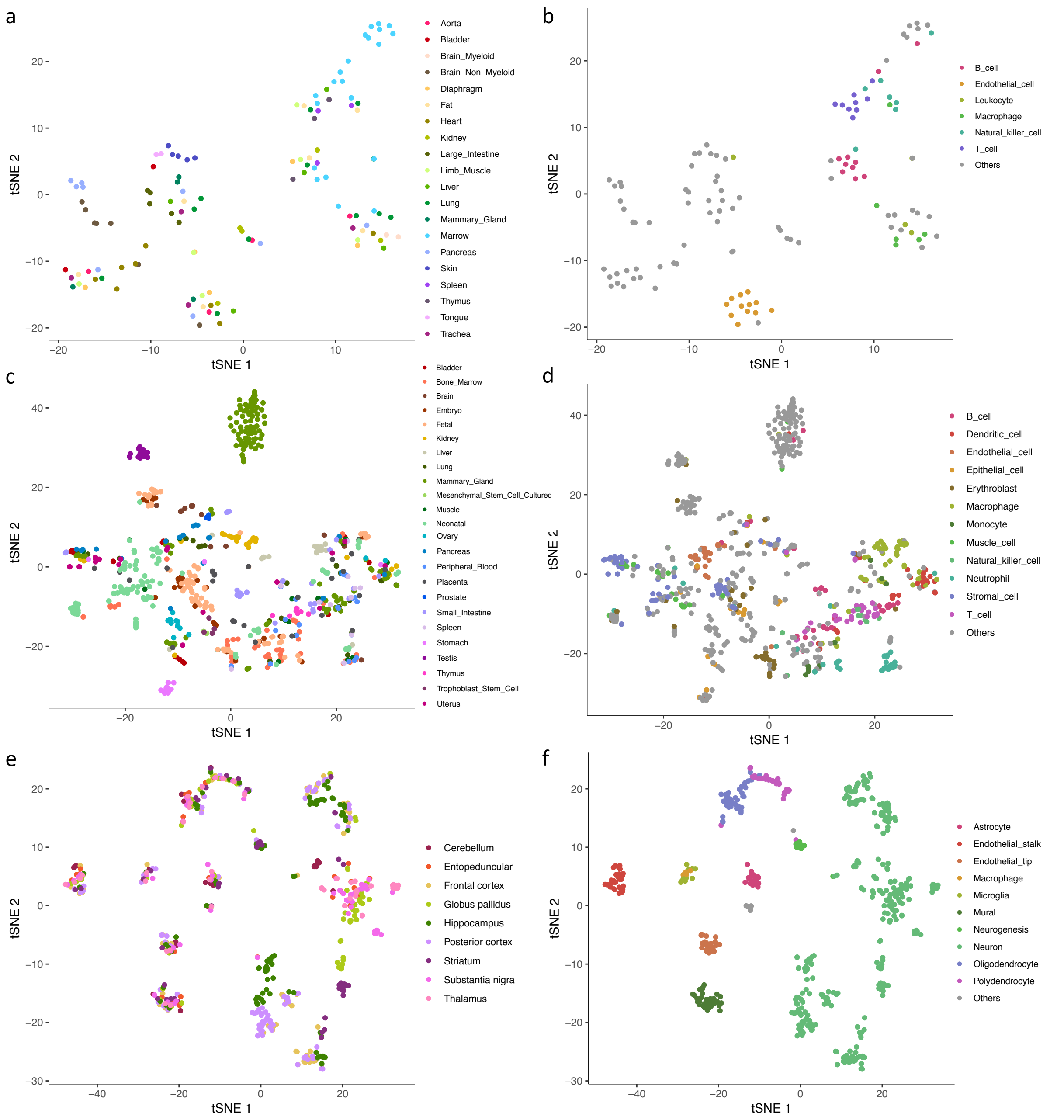

Supplementary Figure 1. 2D projections of cell types from Tabula Muris FACS, Mouse Cell Atlas and DropViz datasets. Each data point represents a cell type in Tabula Muris FACS (a, b), Mouse Cell Atlas (c, d) or DropViz (e, f) datasets and colored by tissue types (a, c), brain regions (e) or general cell types (b, d, f).

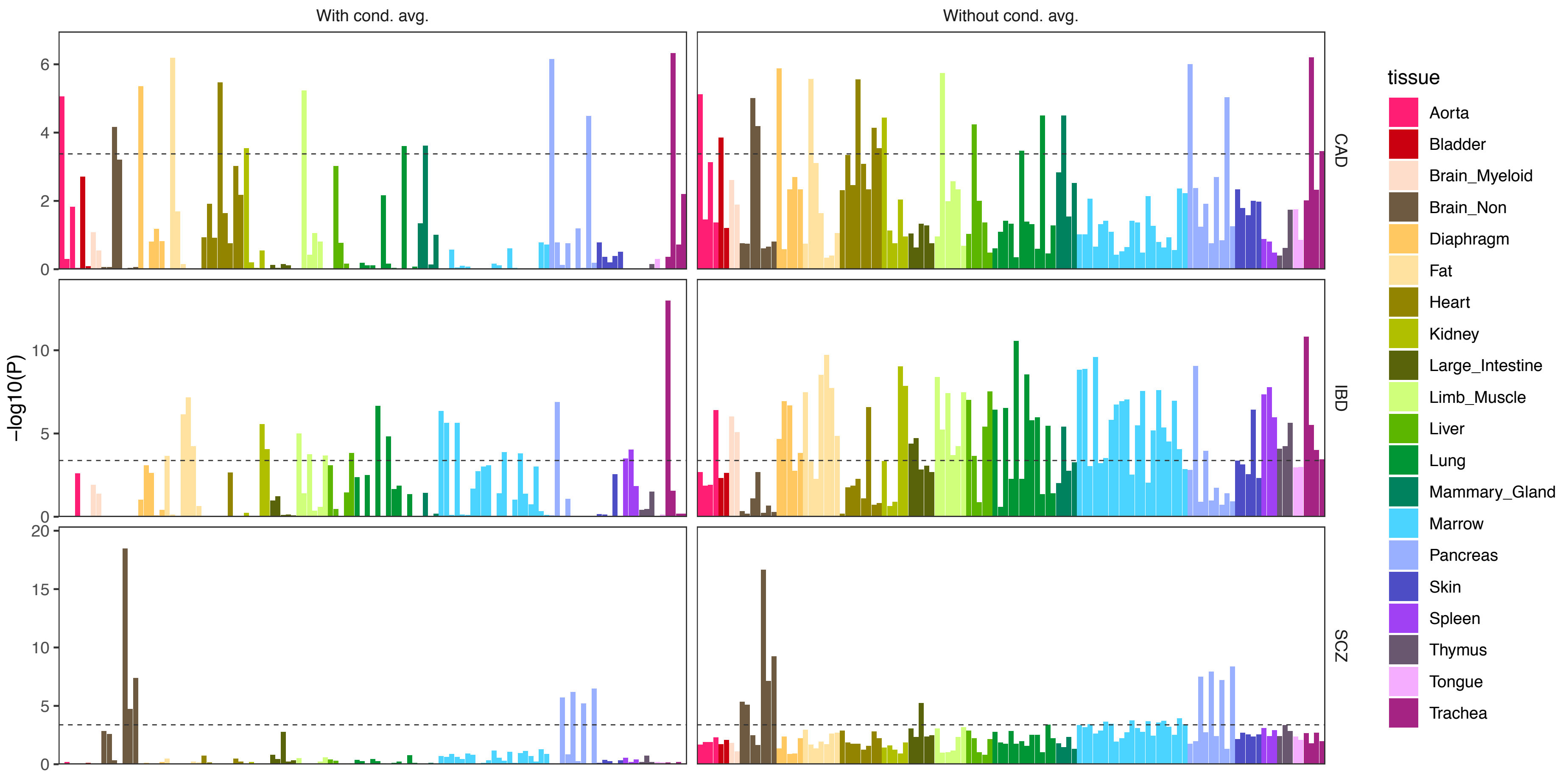

Supplementary Figure 2. MAGMA gene-property analyses with and without conditioning on average expression across cell types. The results of gene-property analyses for CAD, IBD and SCZ GWAS using Tabula Muris FACS dataset. Grey dashed line is Bonferroni corrected P-value (0.05/119). Y-axis is omitted. The left box is without conditioning and the right box is with conditioning on average expression across cell types. Boxes within the same trait are comparable (horizontal).

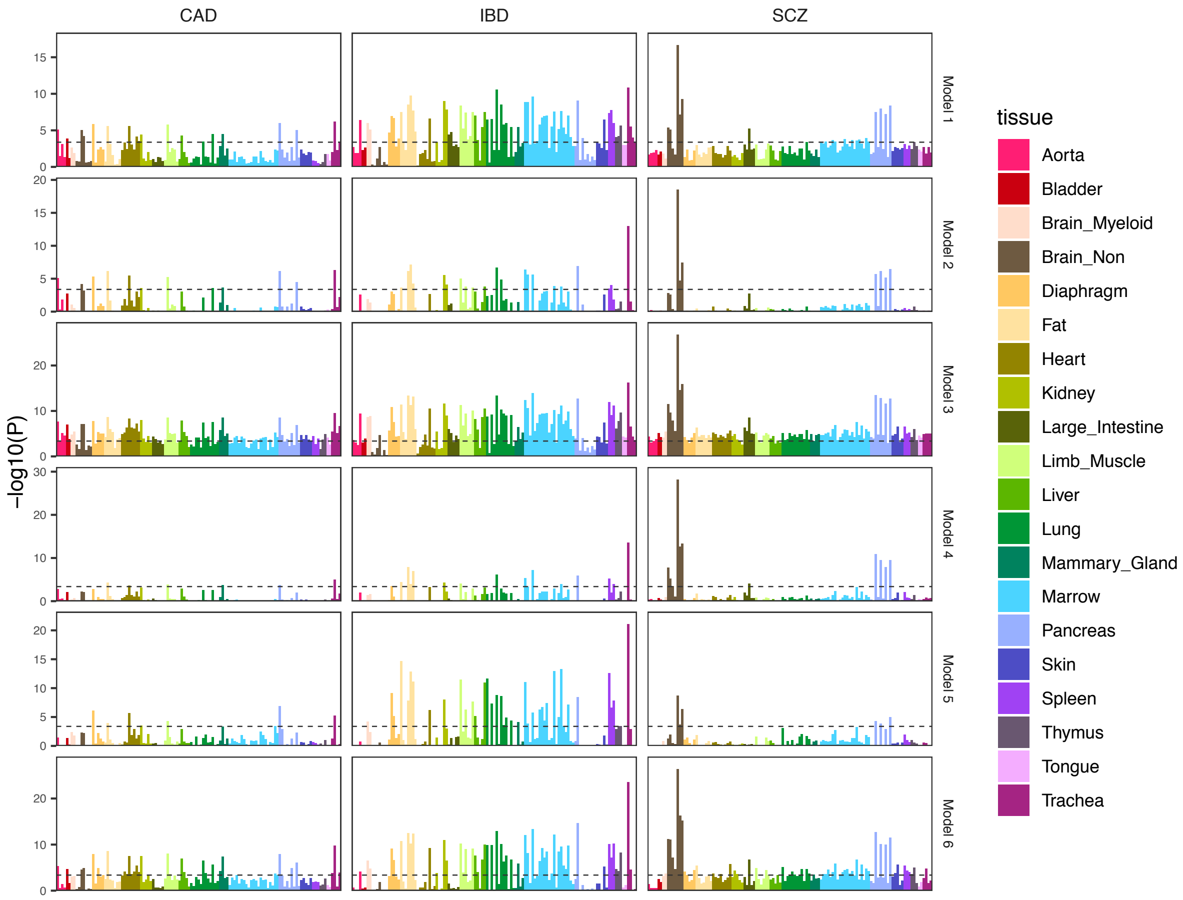

Supplementary Figure 3. The results of gene-property analyses for CAD, IBD and SCZ using Tabula Muris FACS dataset with 6 models described in **Supplementary Note 2**. Model 1: log transformed expression value, Model 2: log transformed expression with conditioning on average across cell types, Model 3: log transformed expression values binned into 40 (+1), Model 4: log transformed expression values binned into 40 (+1) with conditioning on average bin across cell types, Model 5: S score (without binning), and Model 6: S score binned in to 40 (+1). Grey dashed line is Bonferroni corrected P-value (0.05/119). Y-axis is omitted. Boxes within the same trait are comparable (vertical).

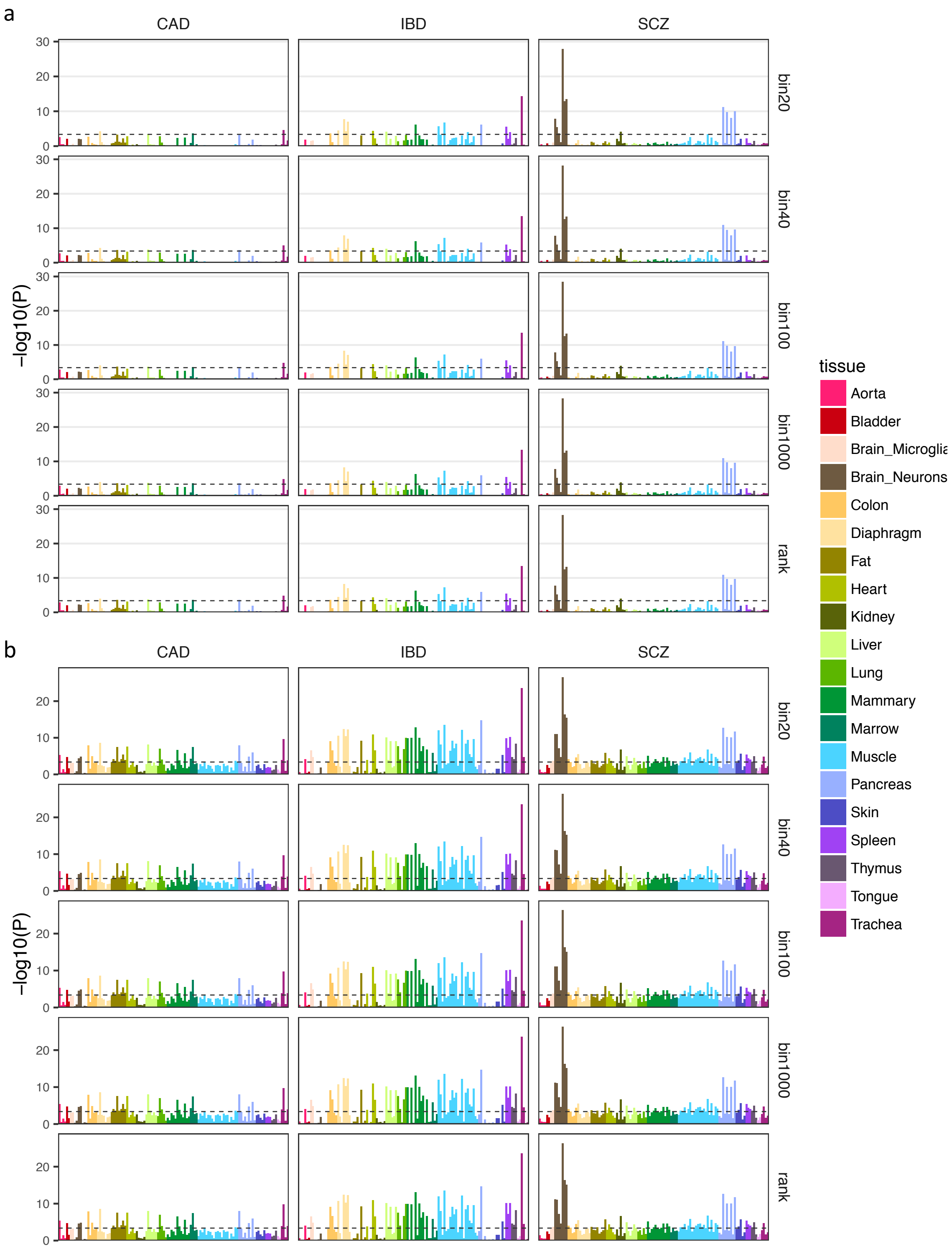

Supplementary Figure 5. Effect of the number of bins. The results of gene-property analyses for CAD, IBD and SCZ GWAS using Tabula Muris FACS dataset with different number of bins. From the top, 20 bins, 40 bins, 100 bins, 1000 bins and full rank (ordered from 1 to the number of genes with non-zero expression) with Model 2 (**a**) and Model 6 (**b**). Grey dashed line is Bonferroni corrected P-value (0.05/119). Y-axis is omitted. Boxes within the same trait are comparable (vertical).

a

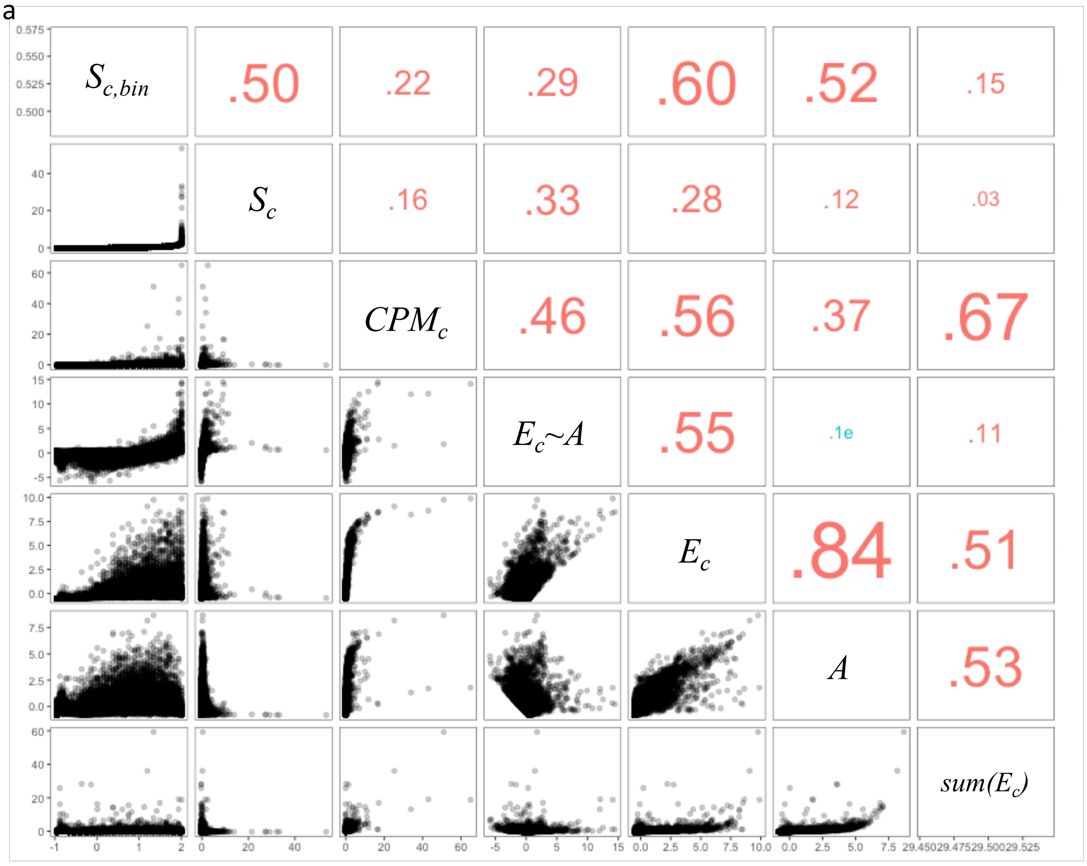

S c,bin
.50
.22
.29
.60
.52
.15
S c
.16
.33
.28
.12
.03
CPM c
.46
.56
.37
.67
E c ~A
.55
.1e
.11
E c
.84
.51
A
.53
sum(E c)

b

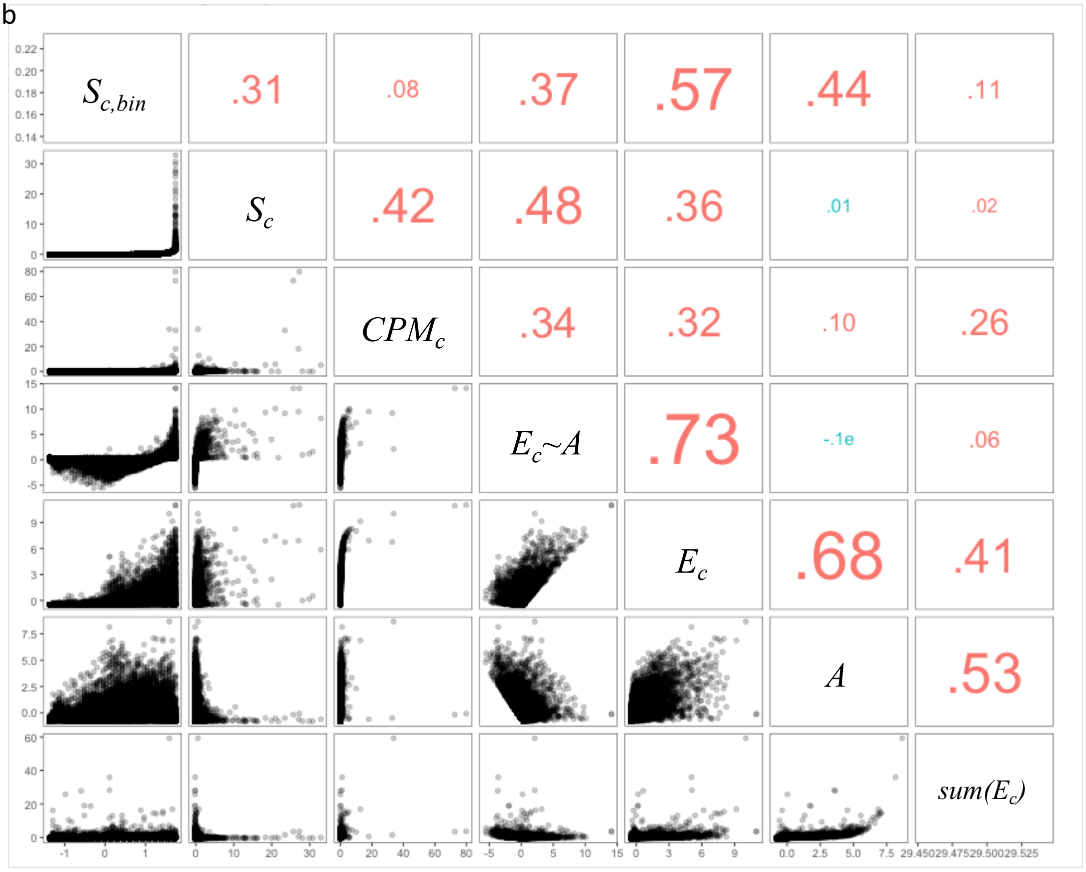

S c,bin
.31
.08
.37
.57
.44
.11
S c
.42
.48
.36
.01
.02
CPM c
.34
.32
.10
.26
E c ~A
.73
-.1e
.06
E c
.68
.41
A
.53
sum(E c)

c

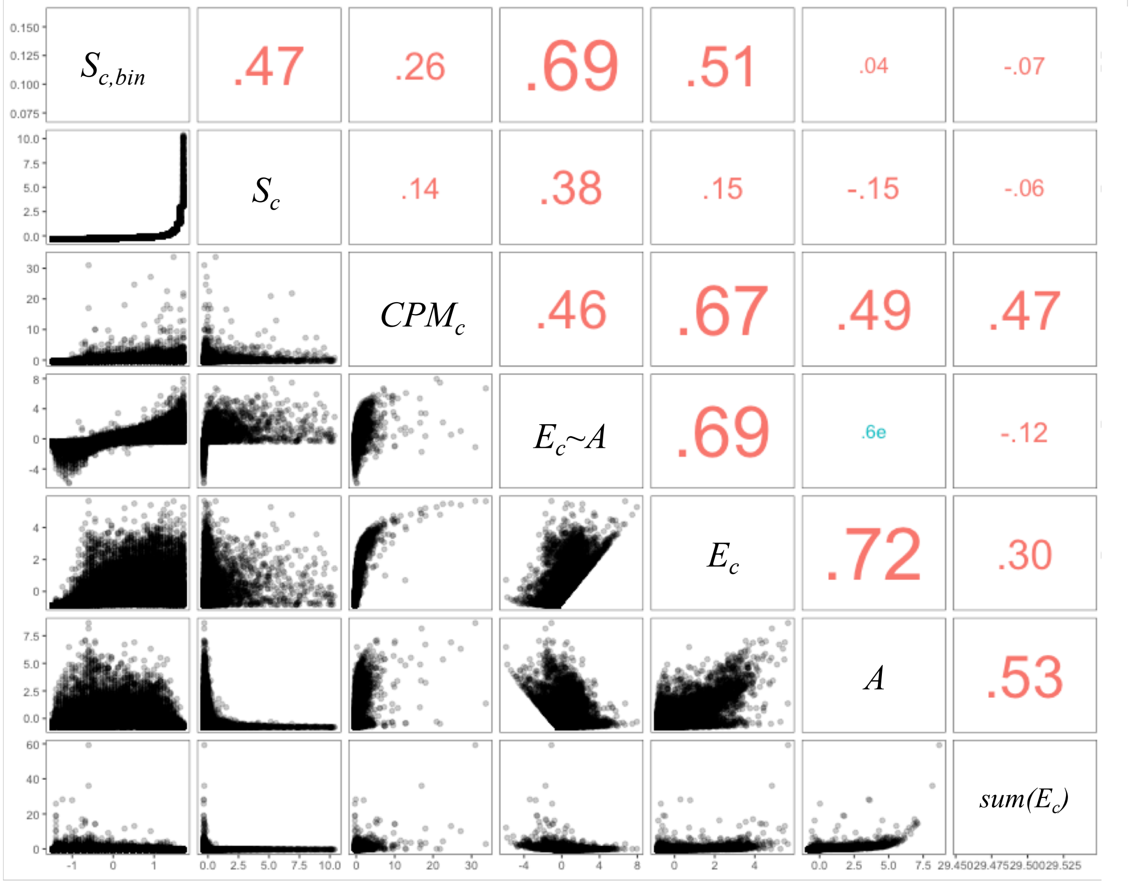

Supplementary Figure 5. Correlation across different expression values. Seven different expression values were compared for 3 cell types; B-cell from lung (**a**), granulocyte from marrow (**b**) and neurons from brain (**c**). Expression values are, *sum*(E_c): sum of expression across cell types, A: average of expression across cell types, E_c: log transformed expression, E_c~A: residuals of regression average expression of cell type c ~ average across cell types, CPM_c: original expression value without log transformation, S_c: S score and $S_{c,bin}$: S score binned into 40 (+1).

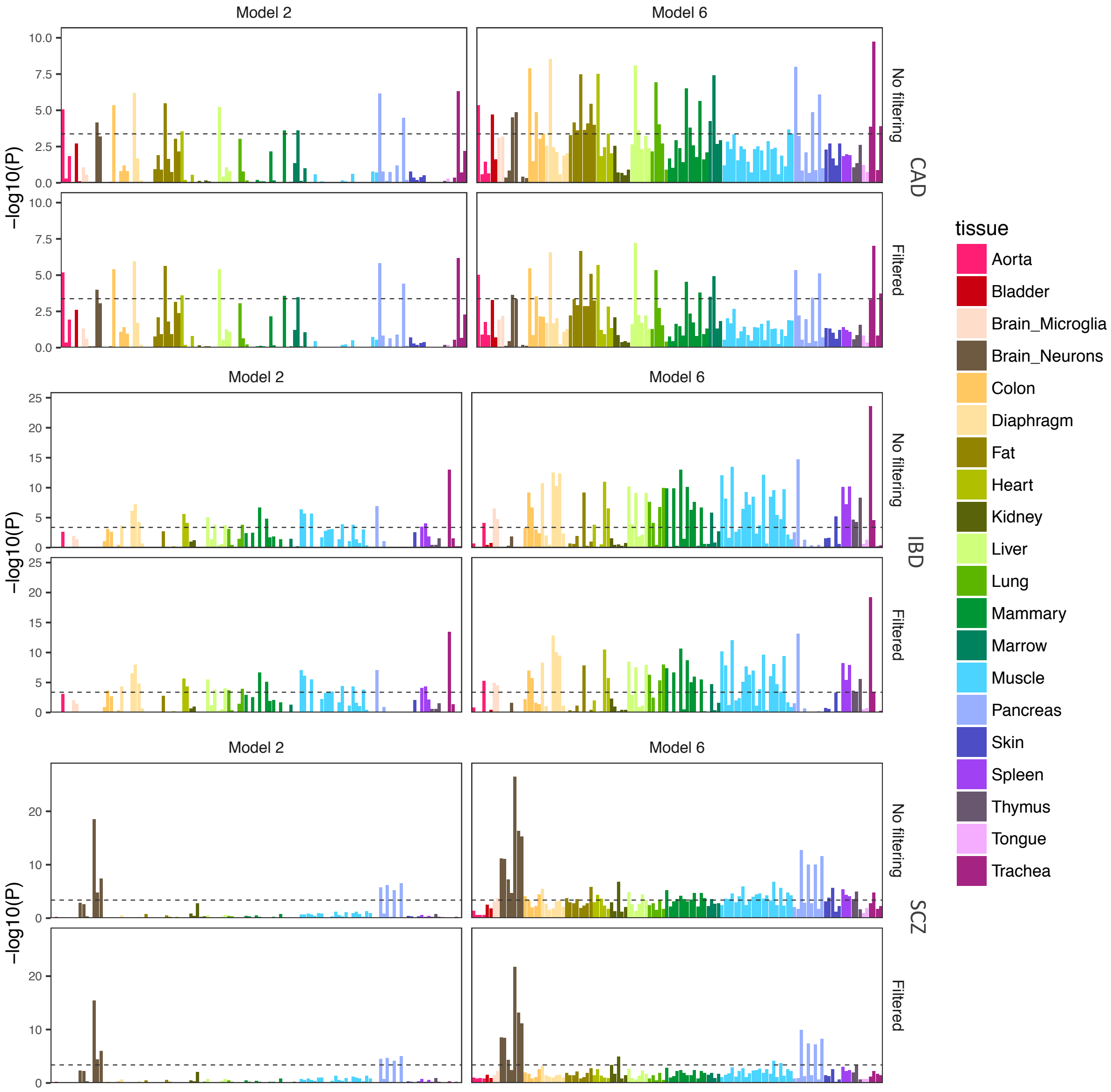

Supplementary Figure 6. Effect of genes with small expression. MAGMA gene-property analyses were performed using Tabula Muris FACS datasets for CAD (top), IBD (middle) and SCZ (bottom) with Model 2 and Model 6. The top panel is without filtering any genes and the bottom panel is filtering genes with zero in >80% of cells in all cell types. Grey dashed line is Bonferroni corrected P-value (0.05/119). Y-axis is omitted. Boxes within the same trait and the same model are comparable (vertical).

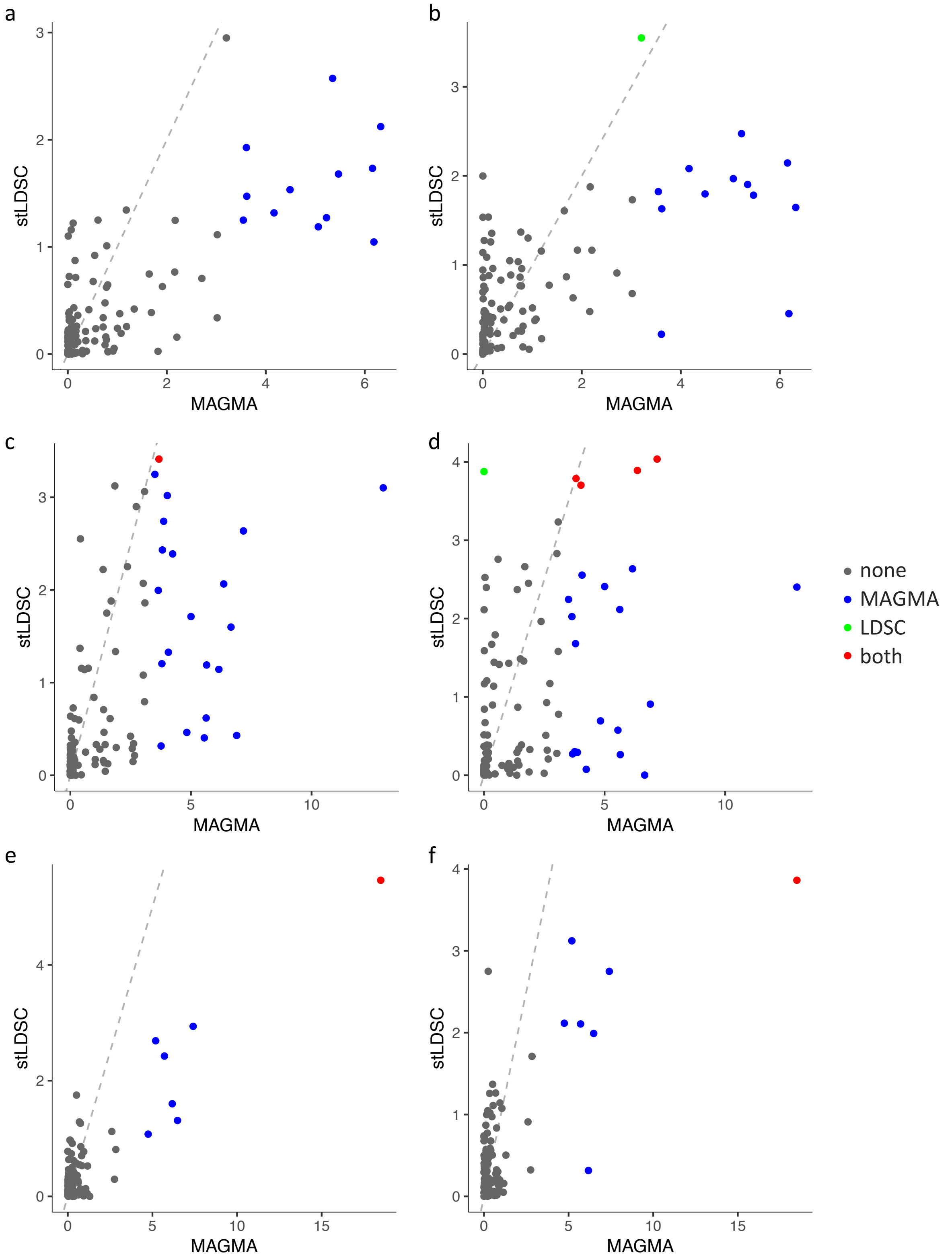

Supplementary Figure 7. Comparison of cell type specificity between MAGMA and LDSC. Axes are -log10 P-value from MAGMA (X-axis) and LDSC (Y-axis). Plot displays cell type association of Tabula Muris FACS datasets with 119 cell types for CAD (**a, b**), IBD (**c, d**) and SCZ (**e, f**). Cell type specific genes for LDSC were defined by S score (**a, c, e**) or residuals after regressing out the average expression across cell types (**b, d, f**). Each data

point represents a cell type and colored by significance after Bonferroni correction (0.05/119).

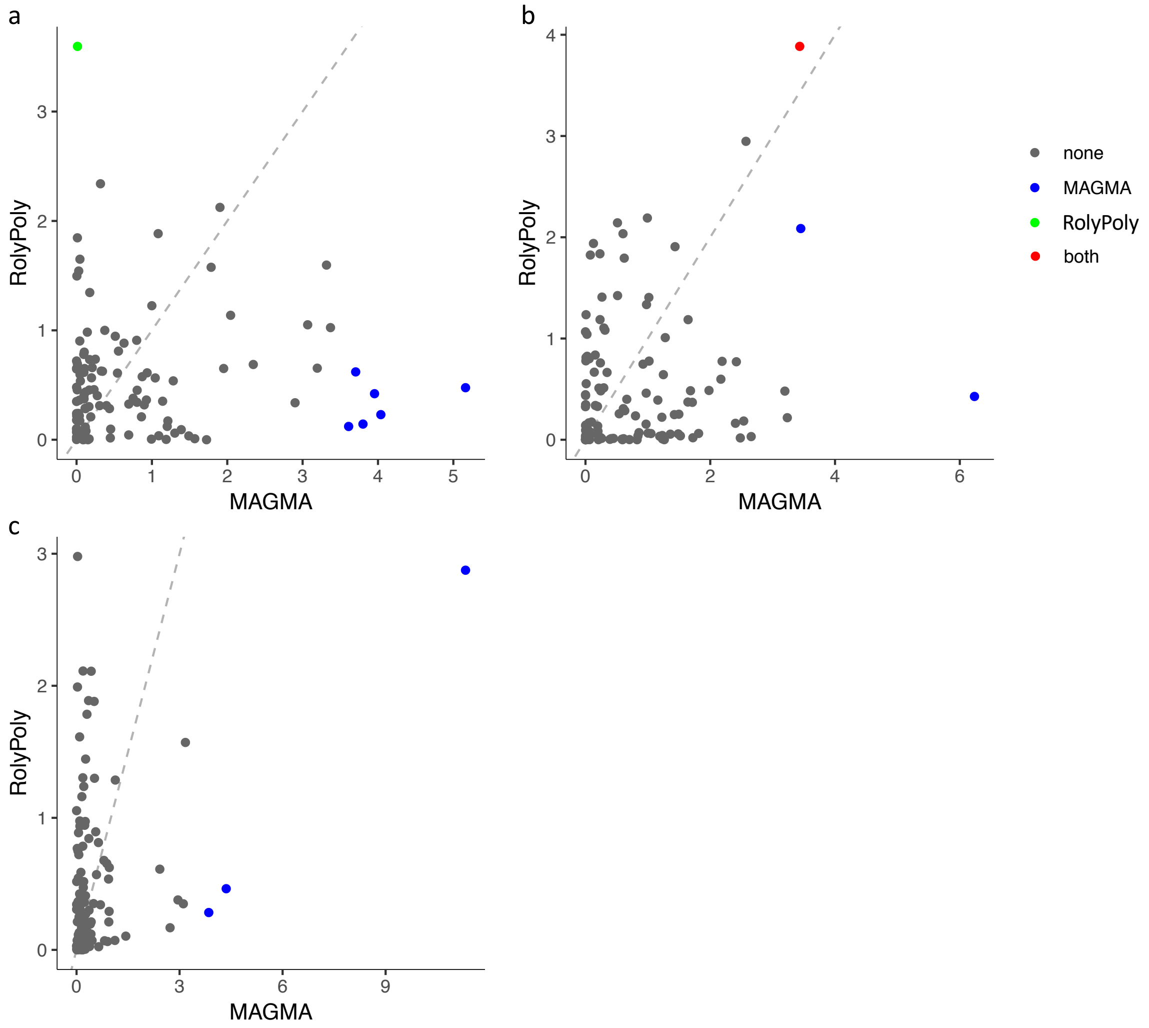

Supplementary Figure 8. Comparison of cell type specificity between MAGMA and RolyPoly. Axes are -log10 P-value from MAGMA (X-axis) and RolyPoly (Y-axis). Plot displays cell type association of Tabula Muris FACS datasets with 119 cell types for CAD (**a**), IBD (**b**) and SCZ (**c**). Each data point represents a cell type and colored by significance after Bonferroni correction (0.05/119).

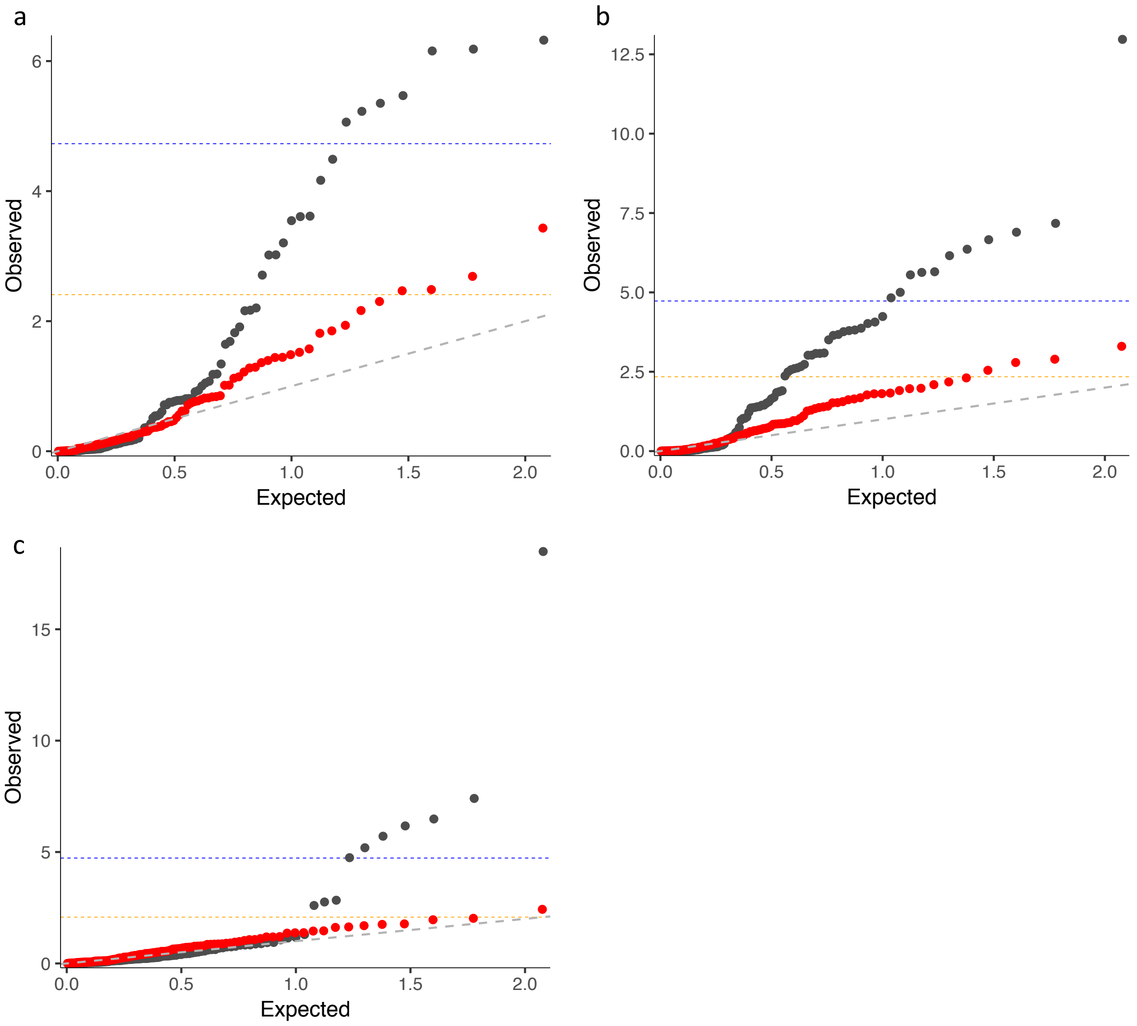

Supplementary Figure 9. QQ plots of associations for 119 cell types from Tabula Muris FACS dataset with CAD (a), IBD (b) and SCZ (c). Grey dots represent marginal P-value of 119 cell types and red dots represent P-value of 118 cell types conditioning on the most significant cell type in the trait. The horizontal blue and orange dashed lines represent Bonferroni P-value threshold and maximum P-value reached FDR<0.05 in a given trait (correcting for all 2,679 cell types across 43 datasets), respectively.

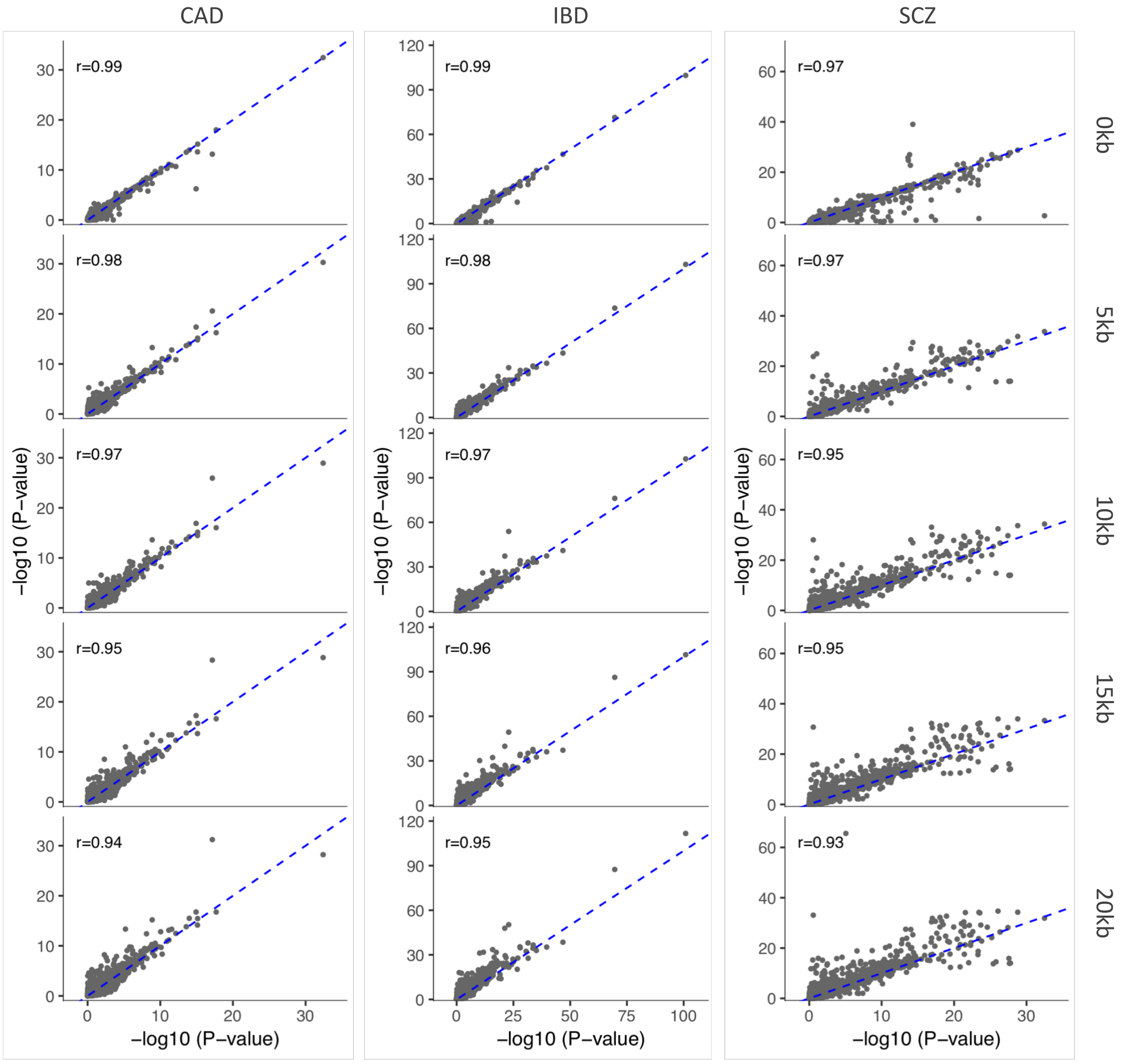

Supplementary Figure 10. Comparison of gene-based P-value with different window sizes. X-axis is -log10 P-value of gene analysis with 1kb window both sides of the genes. Pearson's correlation coefficient is displayed at the left top corner of each plot.

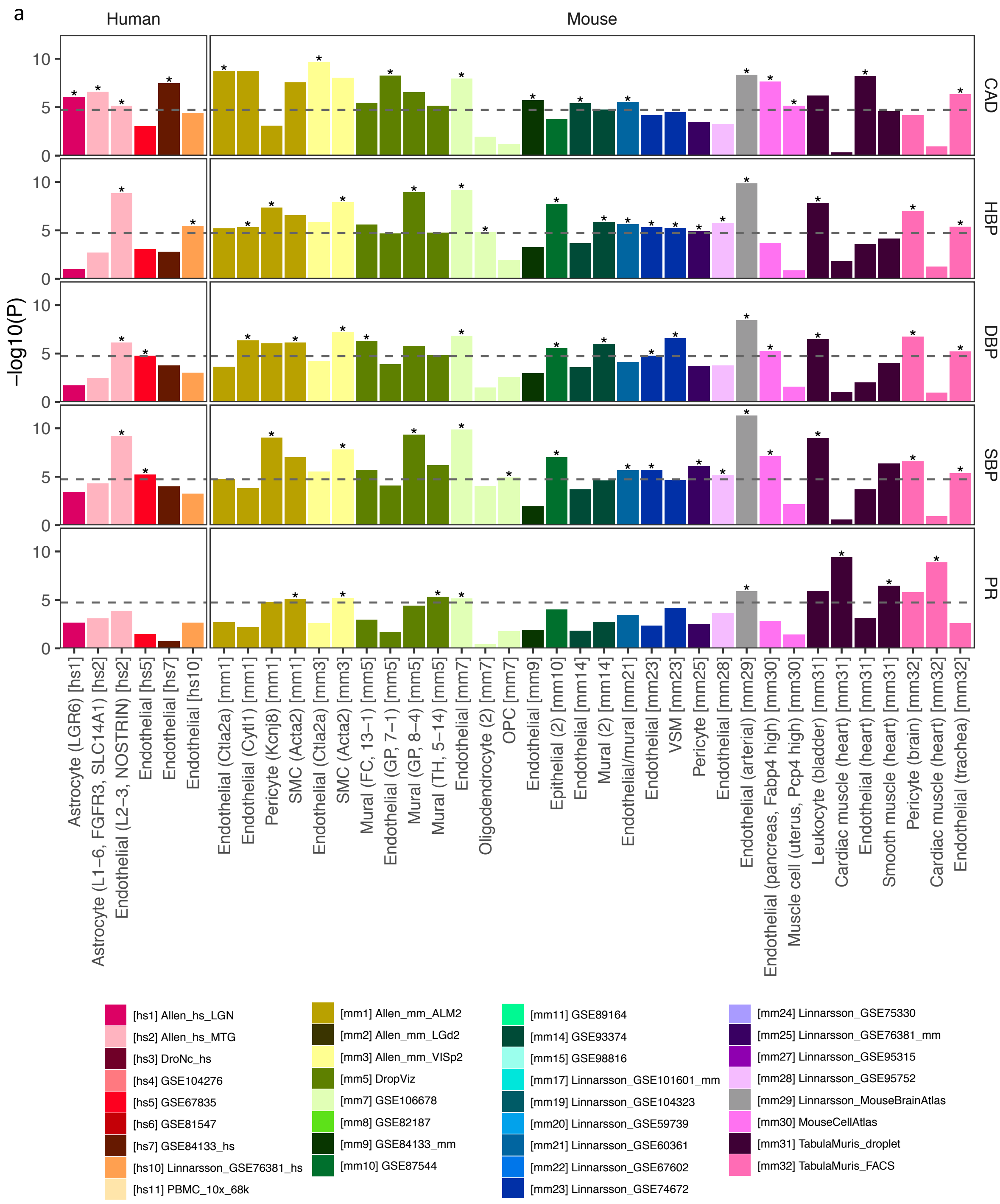
a
Human
Mouse
−log10(P)
0
5
10
CAD
HBP
DBP
SBP
PR
Astrocyte (LGR6) [hs1]
Astrocyte (L1−6, FGFR3, SLC14A1) [hs2]
Endothelial (L2−3, NOSTRIN) [hs2]
Endothelial [hs5]
Endothelial [hs7]
Endothelial [hs10]
Endothelial (Ctla2a) [mm1]
Endothelial (Cytl1) [mm1]
Pericyte (Kcnj8) [mm1]
SMC (Acta2) [mm1]
Endothelial (Ctla2a) [mm3]
SMC (Acta2) [mm3]
Mural (FC, 13−1) [mm5]
Endothelial (GP, 7−1) [mm5]
Mural (GP, 8−4) [mm5]
Mural (TH, 5−14) [mm5]
Endothelial [mm7]
Oligodendrocyte (2) [mm7]
OPC [mm7]
Endothelial [mm9]
Epithelial (2) [mm10]
Endothelial [mm14]
Mural (2) [mm14]
Endothelial/mural [mm21]
Endothelial [mm23]
VSM [mm23]
Pericyte [mm25]
Endothelial [mm28]
Endothelial (arterial) [mm29]
Endothelial (pancreas, Fabp4 high) [mm30]
Muscle cell (uterus, Pcp4 high) [mm30]
Leukocyte (bladder) [mm31]
Cardiac muscle (heart) [mm31]
Endothelial (heart) [mm31]
Smooth muscle (heart) [mm31]
Pericyte (brain) [mm32]
Cardiac muscle (heart) [mm32]
Endothelial (trachea) [mm32]
[hs1] Allen_hs_LGN
[hs2] Allen_hs_MTG
[hs3] DroNc_hs
[hs4] GSE104276
[hs5] GSE67835
[hs6] GSE81547
[hs7] GSE84133_hs
[hs10] Linnarsson_GSE76381_hs
[hs11] PBMC_10x_68k
[mm1] Allen_mm_ALM2
[mm2] Allen_mm_LGd2
[mm3] Allen_mm_VISp2
[mm5] DropViz
[mm7] GSE106678
[mm8] GSE82187
[mm9] GSE84133_mm
[mm10] GSE87544
[mm11] GSE89164
[mm14] GSE93374
[mm15] GSE98816
[mm17] Linnarsson_GSE101601_mm
[mm19] Linnarsson_GSE104323
[mm20] Linnarsson_GSE59739
[mm21] Linnarsson_GSE60361
[mm22] Linnarsson_GSE67602
[mm23] Linnarsson_GSE74672
[mm24] Linnarsson_GSE75330
[mm25] Linnarsson_GSE76381_mm
[mm27] Linnarsson_GSE95315
[mm28] Linnarsson_GSE95752
[mm29] Linnarsson_MouseBrainAtlas
[mm30] MouseCellAtlas
[mm31] TabulaMuris_droplet
[mm32] TabulaMuris_FACS

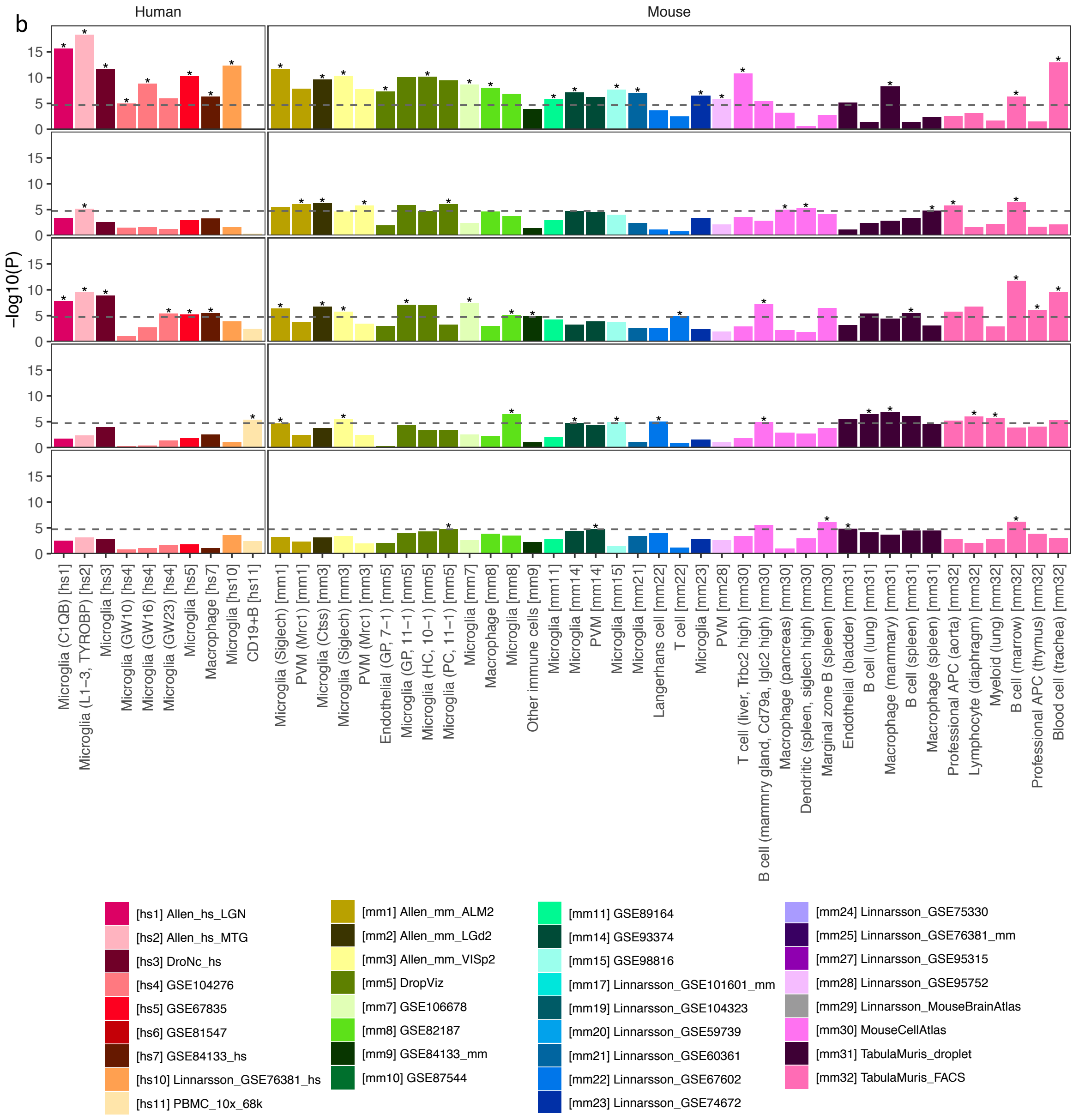
b
Human
Mouse
IBD
MS
RA
SLE
T1D
−log10(P)
[hs1] Allen_hs_LGN
[hs2] Allen_hs_MTG
[hs3] DroNc_hs
[hs4] GSE104276
[hs5] GSE67835
[hs6] GSE81547
[hs7] GSE84133_hs
[hs10] Linnarsson_GSE76381_hs
[hs11] PBMC_10x_68k
[mm1] Allen_mm_ALM2
[mm2] Allen_mm_LGd2
[mm3] Allen_mm_VISp2
[mm5] DropViz
[mm7] GSE106678
[mm8] GSE82187
[mm9] GSE84133_mm
[mm10] GSE87544
[mm11] GSE89164
[mm14] GSE93374
[mm15] GSE98816
[mm17] Linnarsson_GSE101601_mm
[mm19] Linnarsson_GSE104323
[mm20] Linnarsson_GSE59739
[mm21] Linnarsson_GSE60361
[mm22] Linnarsson_GSE67602
[mm23] Linnarsson_GSE74672
[mm24] Linnarsson_GSE75330
[mm25] Linnarsson_GSE76381_mm
[mm27] Linnarsson_GSE95315
[mm28] Linnarsson_GSE95752
[mm29] Linnarsson_MouseBrainAtlas
[mm30] MouseCellAtlas
[mm31] TabulaMuris_droplet
[mm32] TabulaMuris_FACS

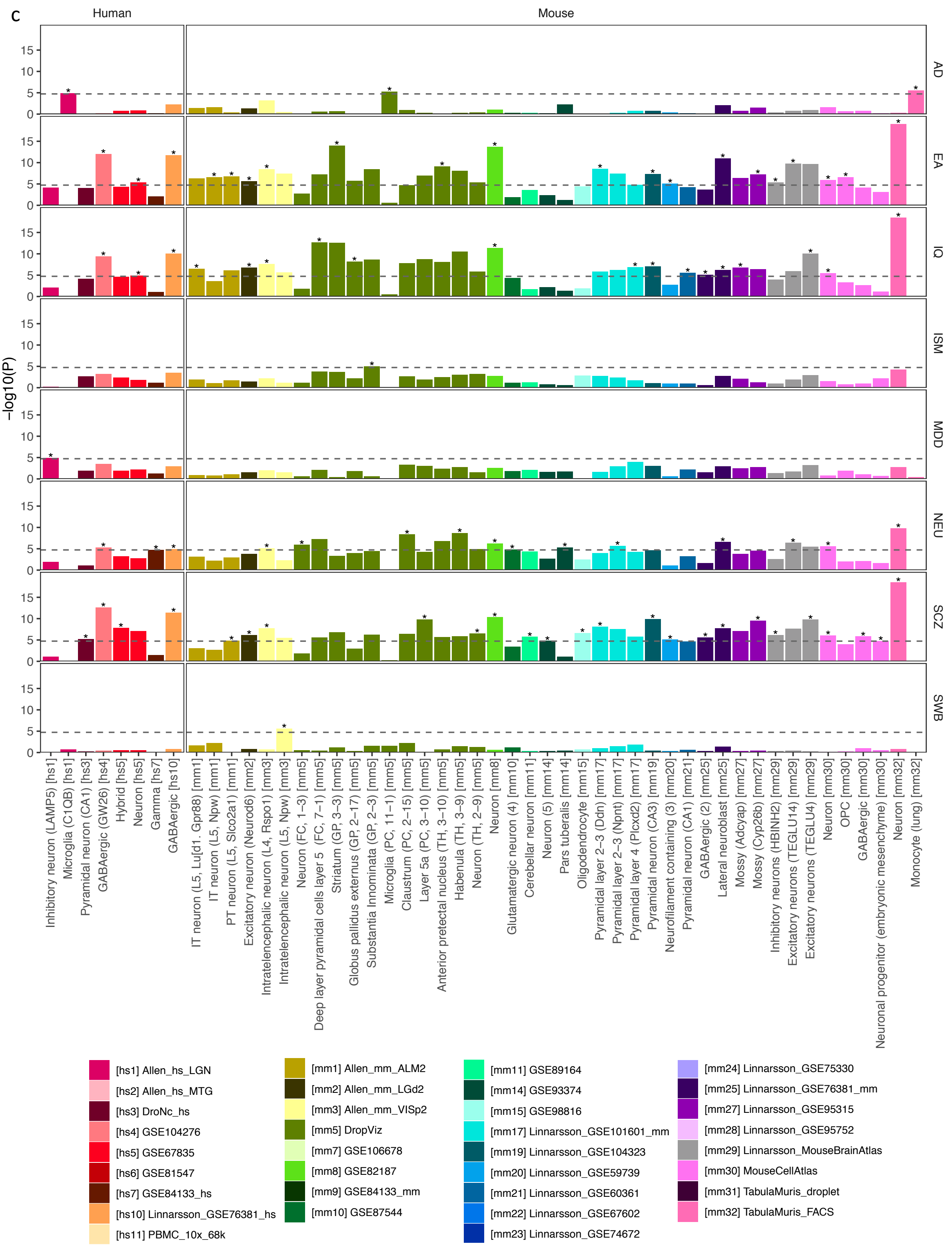
c
Human
Mouse
AD
EA
IQ
ISM
MDD
NEU
SCZ
SWB
−log10(P)
0
5
10
15
Inhibitory neuron (LAMP5) [hs1]
Microglia (C1QB) [hs1]
Pyramidal neuron (CA1) [hs3]
GABAergic (GW26) [hs4]
Hybrid [hs5]
Neuron [hs5]
Gamma [hs7]
GABAergic [hs10]
IT neuron (L5, Lu[d1. Gpr88) [mm1]
IT neuron (L5, Npw) [mm1]
PT neuron (L5, Slco2a1) [mm1]
Excitatory neuron (Neurod6) [mm2]
Intratelencephalic neuron (L4, Rspo1) [mm3]
Intratelencephalic neuron (L5, Npw) [mm3]
Neuron (FC, 1−3) [mm5]
Deep layer pyramidal cells layer 5 (FC, 7−1) [mm5]
Striatum (GP, 3−3) [mm5]
Globus pallidus externus (GP, 2−17) [mm5]
Substantia Innominata (GP, 2−3) [mm5]
Microglia (PC, 11−1) [mm5]
Claustrum (PC, 2−15) [mm5]
Layer 5a (PC, 3−10) [mm5]
Anterior pretectal nucleus (TH, 3−10) [mm5]
Habenula (TH, 3−9) [mm5]
Neuron (TH, 2−9) [mm5]
Neuron [mm8]
Glutamatergic neuron (4) [mm10]
Cerebellar neuron [mm11]
Neuron (5) [mm14]
Pars tuberalis [mm14]
Oligodendrocyte [mm15]
Pyramidal layer 2−3 (Ddn) [mm17]
Pyramidal layer 2−3 (Npnt) [mm17]
Pyramidal layer 4 (Plcxd2) [mm17]
Pyramidal neuron (CA3) [mm19]
Neurofilament containing (3) [mm20]
Pyramidal neuron (CA1) [mm21]
GABAergic (2) [mm25]
Lateral neuroblast [mm25]
Mossy (Adcyap) [mm27]
Mossy (Cyp26b) [mm27]
Inhibitory neurons (HBINH2) [mm29]
Excitatory neurons (TEGLU14) [mm29]
Excitatory neurons (TEGLU4) [mm29]
Neuron [mm30]
OPC [mm30]
GABAergic [mm30]
Neuronal progenitor (embryonic mesenchyme) [mm30]
Neuron [mm32]
Monocyte (lung) [mm32]
[hs1] Allen_hs_LGN
[hs2] Allen_hs_MTG
[hs3] DroNc_hs
[hs4] GSE104276
[hs5] GSE67835
[hs6] GSE81547
[hs7] GSE84133_hs
[hs10] Linnarsson_GSE76381_hs
[hs11] PBMC_10x_68k
[mm1] Allen_mm_ALM2
[mm2] Allen_mm_LGd2
[mm3] Allen_mm_VISp2
[mm5] DropViz
[mm7] GSE106678
[mm8] GSE82187
[mm9] GSE84133_mm
[mm10] GSE87544
[mm11] GSE89164
[mm14] GSE93374
[mm15] GSE98816
[mm17] Linnarsson_GSE101601_mm
[mm19] Linnarsson_GSE104323
[mm20] Linnarsson_GSE59739
[mm21] Linnarsson_GSE60361
[mm22] Linnarsson_GSE67602
[mm23] Linnarsson_GSE74672
[mm24] Linnarsson_GSE75330
[mm25] Linnarsson_GSE76381_mm
[mm27] Linnarsson_GSE95315
[mm28] Linnarsson_GSE95752
[mm29] Linnarsson_MouseBrainAtlas
[mm30] MouseCellAtlas
[mm31] TabulaMuris_droplet
[mm32] TabulaMuris_FACS

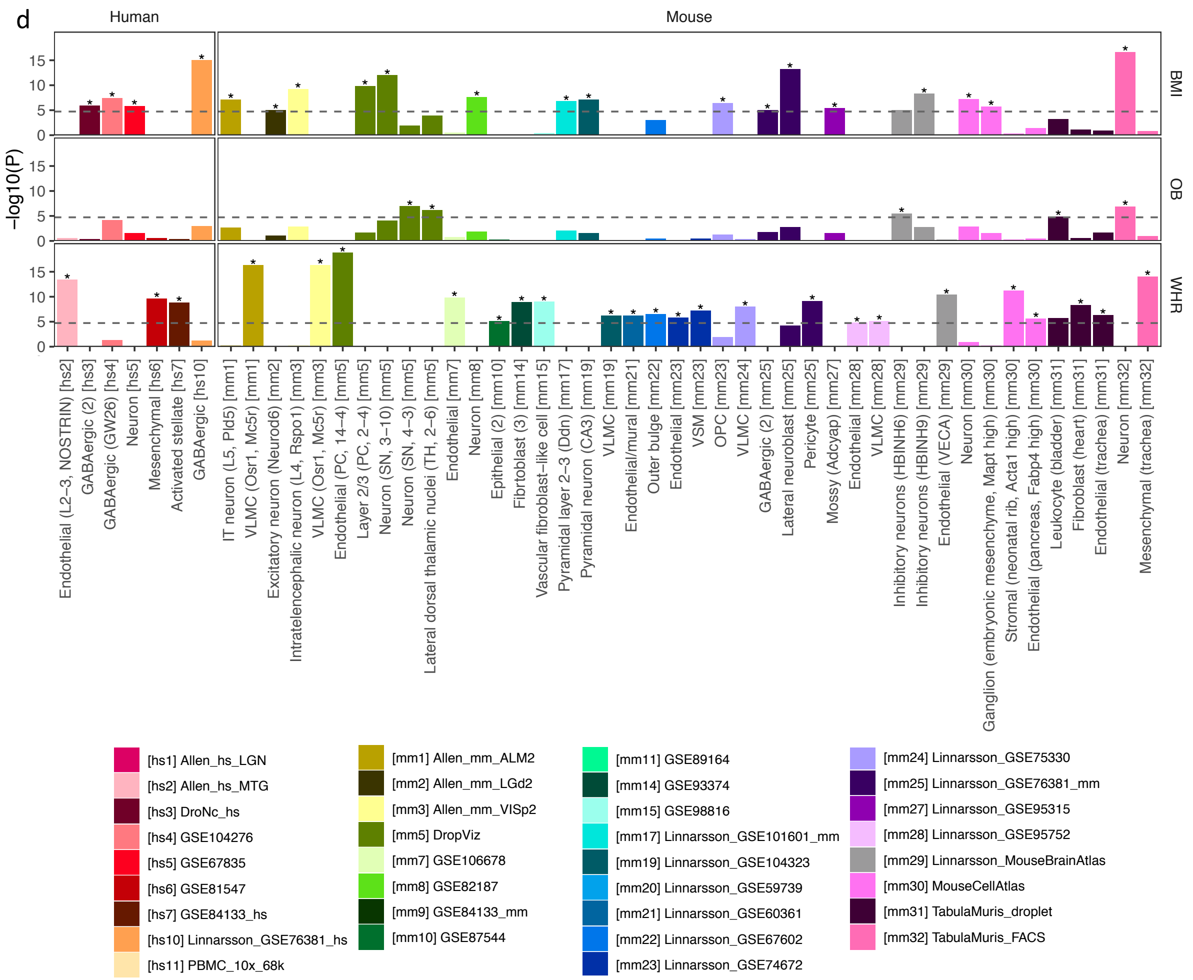

Supplementary Figure 11. Independent significant associations of cell types (from step 2). The plot displays only traits with at least one significant cell type association (21 out of 26 traits). For each domain (or domain cluster), cell types that are retained after step 2 (per dataset conditional analysis) in at least one of the traits are displayed for cardiovascular (**a**), immunological (**b**), cognitive, neurological and psychiatric (**c**), and metabolic (**d**) domains. Horizontal dashed lines represent the Bonferroni correction threshold (0.05/2679) and stars indicate cell types retained from step 2 for the corresponding trait. Bars are colored by datasets. Cell types are labeled using their common name with additional information in parentheses (which is needed when referring back to the label from the original study). The index of the dataset is in square brackets. For traits, CAD: coronary artery disease, HBP: high blood pressure, DBP: diastolic blood pressure, SBP: systolic blood pressure, PR: pulse rate, IBD: inflammatory bowel disease, MS: multiple sclerosis, RA: rheumatoid arthritis, SLE: systemic lupus erythematosus, T1D: type 1 diabetes, AD: Alzheimer disease, EA: educational attainment, IQ: intelligence, ISM: insomnia, MDD: major depressive disorder, NEU: neuroticism, SCZ: schizophrenia, SWB: subjective well-being, BMI: body mass index, OB: obesity, WHR: waist hip ratio. For cell types, APC: antigen presenting cell, FC: frontal cortex, GP: globus pallidus, IT: intratelencephalic, L: layer, OPC: oligodendrocyte precursor cell, PC: posterior cortex, PT: pyramidal tract, PVM: perivascular macrophage, SMC: smooth muscle cell, SN: substantia nigra, TH: thalamus, VLMC: vascular leptomeningeal cell, VSM: vascular smooth muscle.

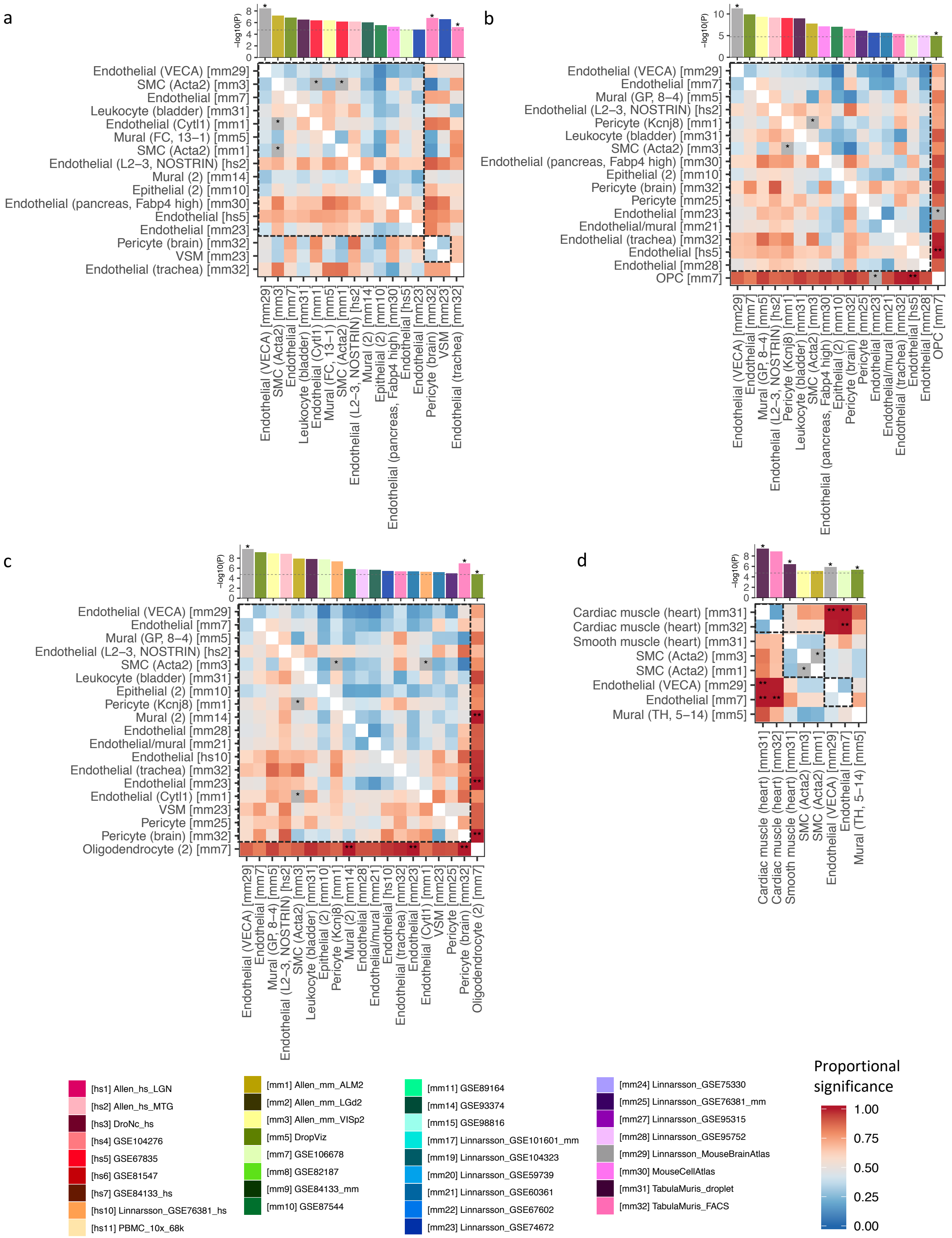
a
b
c
d
−log10(P)
Endothelial (VECA) [mm29]
SMC (Acta2) [mm3]
Endothelial [mm7]
Leukocyte (bladder) [mm31]
Endothelial (Cytl1) [mm1]
Mural (FC, 13−1) [mm5]
SMC (Acta2) [mm1]
Endothelial (L2−3, NOSTRIN) [hs2]
Mural (2) [mm14]
Epithelial (2) [mm10]
Endothelial (pancreas, Fabp4 high) [mm30]
Endothelial [hs5]
Endothelial [mm23]
Pericyte (brain) [mm32]
VSM [mm23]
Endothelial (trachea) [mm32]
Mural (GP, 8−4) [mm5]
Pericyte (Kcnj8) [mm1]
Pericyte [mm25]
Endothelial/mural [mm21]
Endothelial [mm28]
OPC [mm7]
Endothelial [hs10]
Oligodendrocyte (2) [mm7]
Cardiac muscle (heart) [mm31]
Cardiac muscle (heart) [mm32]
Smooth muscle (heart) [mm31]
Mural (TH, 5−14) [mm5]
[hs1] Allen_hs_LGN
[hs2] Allen_hs_MTG
[hs3] DroNc_hs
[hs4] GSE104276
[hs5] GSE67835
[hs6] GSE81547
[hs7] GSE84133_hs
[hs10] Linnarsson_GSE76381_hs
[hs11] PBMC_10x_68k
[mm1] Allen_mm_ALM2
[mm2] Allen_mm_LGd2
[mm3] Allen_mm_VISp2
[mm5] DropViz
[mm7] GSE106678
[mm8] GSE82187
[mm9] GSE84133_mm
[mm10] GSE87544
[mm11] GSE89164
[mm14] GSE93374
[mm15] GSE98816
[mm17] Linnarsson_GSE101601_mm
[mm19] Linnarsson_GSE104323
[mm20] Linnarsson_GSE59739
[mm21] Linnarsson_GSE60361
[mm22] Linnarsson_GSE67602
[mm23] Linnarsson_GSE74672
[mm24] Linnarsson_GSE75330
[mm25] Linnarsson_GSE76381_mm
[mm27] Linnarsson_GSE95315
[mm28] Linnarsson_GSE95752
[mm29] Linnarsson_MouseBrainAtlas
[mm30] MouseCellAtlas
[mm31] TabulaMuris_droplet
[mm32] TabulaMuris_FACS
Proportional significance
1.00
0.75
0.50
0.25
0.00

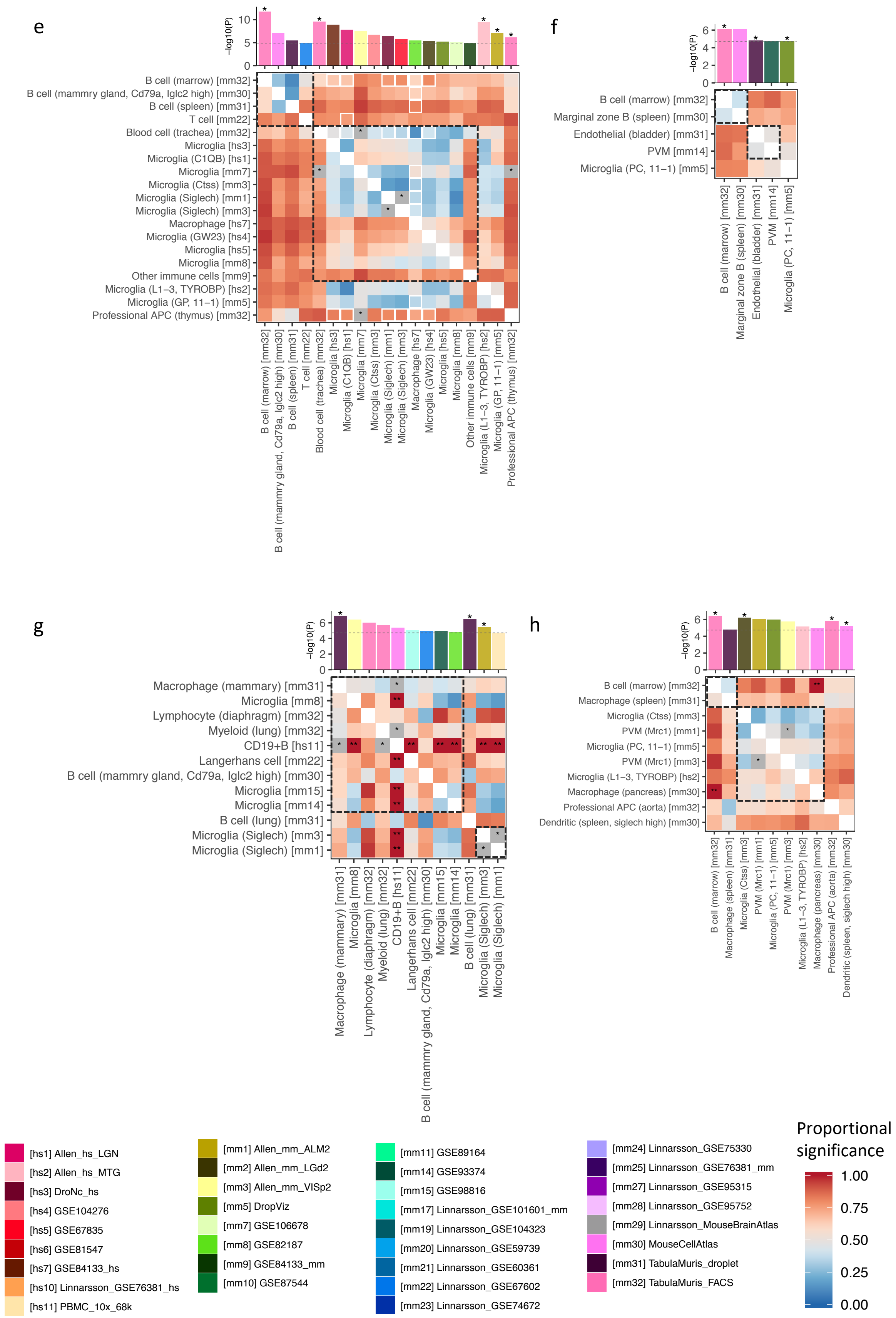
e
−log10(P)
B cell (marrow) [mm32]
B cell (mammry gland, Cd79a, Iglc2 high) [mm30]
B cell (spleen) [mm31]
T cell [mm22]
Blood cell (trachea) [mm32]
Microglia [hs3]
Microglia (C1QB) [hs1]
Microglia [mm7]
Microglia (Ctss) [mm3]
Microglia (Siglech) [mm1]
Microglia (Siglech) [mm3]
Macrophage [hs7]
Microglia (GW23) [hs4]
Microglia [hs5]
Microglia [mm8]
Other immune cells [mm9]
Microglia (L1−3, TYROBP) [hs2]
Microglia (GP, 11−1) [mm5]
Professional APC (thymus) [mm32]
f
−log10(P)
B cell (marrow) [mm32]
Marginal zone B (spleen) [mm30]
Endothelial (bladder) [mm31]
PVM [mm14]
Microglia (PC, 11−1) [mm5]
g
−log10(P)
Macrophage (mammary) [mm31]
Microglia [mm8]
Lymphocyte (diaphragm) [mm32]
Myeloid (lung) [mm32]
CD19+B [hs11]
Langerhans cell [mm22]
B cell (mammry gland, Cd79a, Iglc2 high) [mm30]
Microglia [mm15]
Microglia [mm14]
B cell (lung) [mm31]
Microglia (Siglech) [mm3]
Microglia (Siglech) [mm1]
h
−log10(P)
B cell (marrow) [mm32]
Macrophage (spleen) [mm31]
Microglia (Ctss) [mm3]
PVM (Mrc1) [mm1]
Microglia (PC, 11−1) [mm5]
PVM (Mrc1) [mm3]
Microglia (L1−3, TYROBP) [hs2]
Macrophage (pancreas) [mm30]
Professional APC (aorta) [mm32]
Dendritic (spleen, siglech high) [mm30]
[hs1] Allen_hs_LGN
[hs2] Allen_hs_MTG
[hs3] DroNc_hs
[hs4] GSE104276
[hs5] GSE67835
[hs6] GSE81547
[hs7] GSE84133_hs
[hs10] Linnarsson_GSE76381_hs
[hs11] PBMC_10x_68k
[mm1] Allen_mm_ALM2
[mm2] Allen_mm_LGd2
[mm3] Allen_mm_VISp2
[mm5] DropViz
[mm7] GSE106678
[mm8] GSE82187
[mm9] GSE84133_mm
[mm10] GSE87544
[mm11] GSE89164
[mm14] GSE93374
[mm15] GSE98816
[mm17] Linnarsson_GSE101601_mm
[mm19] Linnarsson_GSE104323
[mm20] Linnarsson_GSE59739
[mm21] Linnarsson_GSE60361
[mm22] Linnarsson_GSE67602
[mm23] Linnarsson_GSE74672
[mm24] Linnarsson_GSE75330
[mm25] Linnarsson_GSE76381_mm
[mm27] Linnarsson_GSE95315
[mm28] Linnarsson_GSE95752
[mm29] Linnarsson_MouseBrainAtlas
[mm30] MouseCellAtlas
[mm31] TabulaMuris_droplet
[mm32] TabulaMuris_FACS
Proportional significance
1.00
0.75
0.50
0.25
0.00

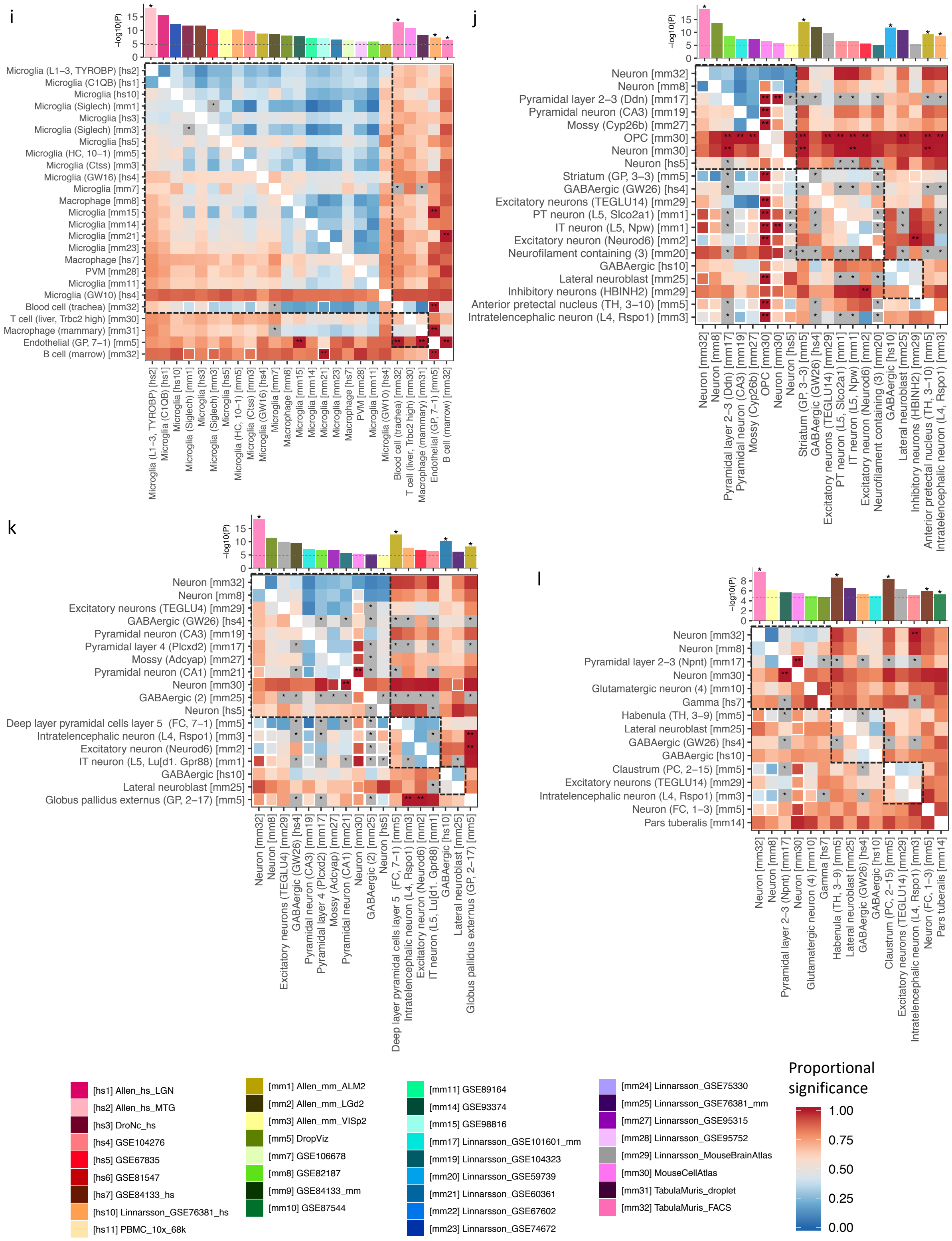

i
j
k
l
−log10(P)
[hs1] Allen_hs_LGN
[hs2] Allen_hs_MTG
[hs3] DroNc_hs
[hs4] GSE104276
[hs5] GSE67835
[hs6] GSE81547
[hs7] GSE84133_hs
[hs10] Linnarsson_GSE76381_hs
[hs11] PBMC_10x_68k
[mm1] Allen_mm_ALM2
[mm2] Allen_mm_LGd2
[mm3] Allen_mm_VISp2
[mm5] DropViz
[mm7] GSE106678
[mm8] GSE82187
[mm9] GSE84133_mm
[mm10] GSE87544
[mm11] GSE89164
[mm14] GSE93374
[mm15] GSE98816
[mm17] Linnarsson_GSE101601_mm
[mm19] Linnarsson_GSE104323
[mm20] Linnarsson_GSE59739
[mm21] Linnarsson_GSE60361
[mm22] Linnarsson_GSE67602
[mm23] Linnarsson_GSE74672
[mm24] Linnarsson_GSE75330
[mm25] Linnarsson_GSE76381_mm
[mm27] Linnarsson_GSE95315
[mm28] Linnarsson_GSE95752
[mm29] Linnarsson_MouseBrainAtlas
[mm30] MouseCellAtlas
[mm31] TabulaMuris_droplet
[mm32] TabulaMuris_FACS
Proportional significance
1.00
0.75
0.50
0.25
0.00

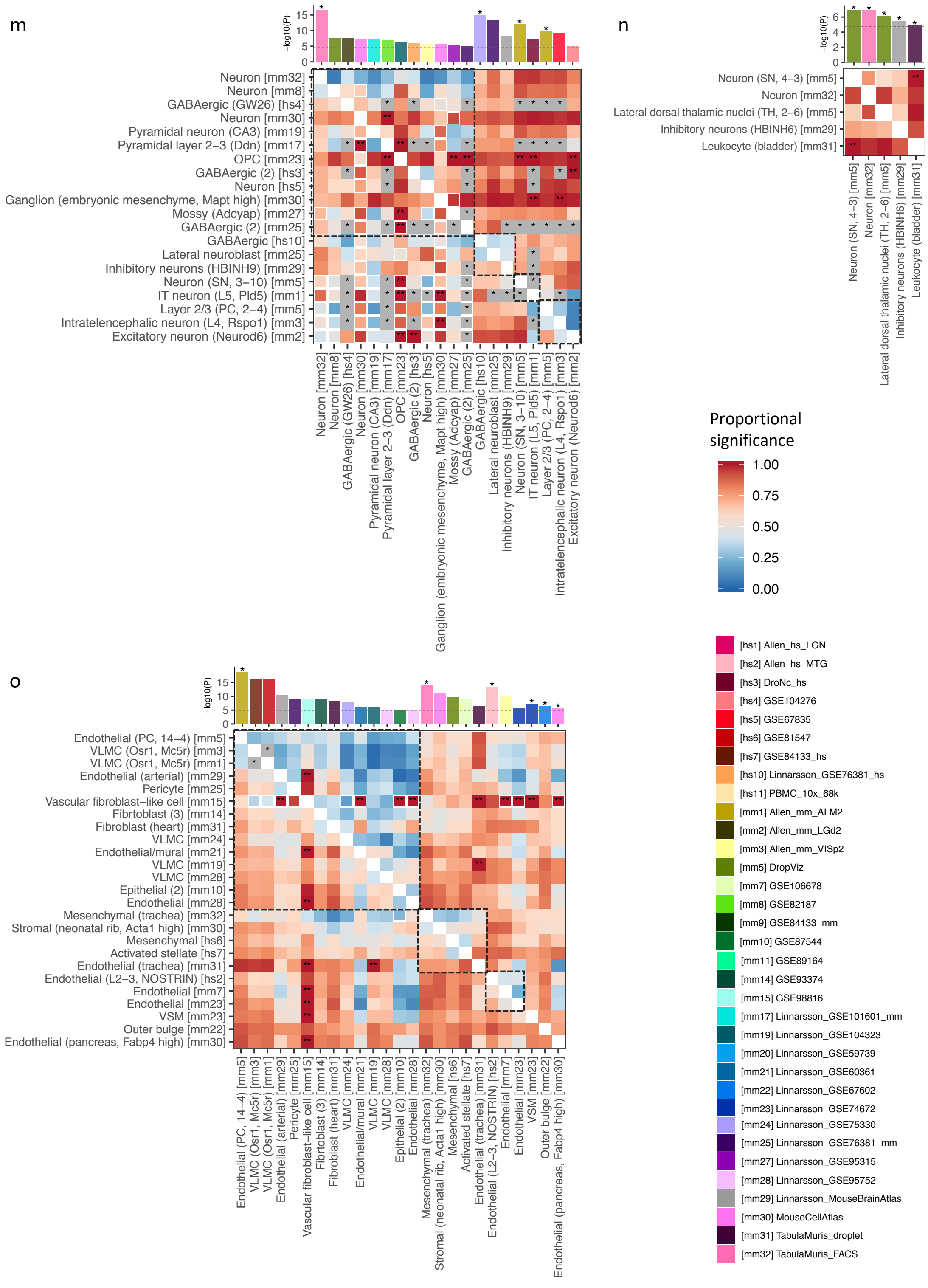

Supplementary Figure 12. Heatmap of pair-wise cross-datasets conditional analyses (step 3) for cell types retained from the step 2. Only traits with more than 3 cell types retained from step 2 are displayed. Coronary artery disease and schizophrenia are displayed in **Fig. 5** in the main text. Cell types are labeled using their common name with additional information in parentheses (which is needed when referring back to the label from the original study). The index of the dataset is in square brackets. The heatmap is asymmetric; a cell on row *i* and column *j* is cross-datasets (CD) proportional significance (PS) of cell type *j* conditioning on cell type *i*. The CD PS is computed as -log10(CD conditional P-value)/-

log10(CD marginal P-value) The size of the square is smaller (80%) when 50% of the marginal association of a cell type in column *j* is explained by adding the average expression of the dataset in row *i* (before conditioning on the expression of cell type *i*). Stars on the heatmap represent pair of cell types that are colinear. Double starts on the heatmap represent CD PS>1. The bar plot at the top illustrates marginal P-value of the cell types on x-axis and stars represent independently associated cell types. Cell types are clustered by their independence, and within each cluster cell types are ordered by their marginal P-value. Stars represent independently associated cell types. (**a**) diastolic blood pressure, (**b**) systolic blood pressure, (**c**) high blood pressure, (**d**) pulse rate, (**e**) rheumatoid arthritis, (**f**) type 1 diabetes, (**g**) systemic lupus erythematosus, (**h**) multiple sclerosis, (**i**) inflammatory bowel disease, (**j**) educational attainment, (**k**) intelligence, (**l**) neuroticism, (**m**) body mass index, (**n**) obesity, (**o**) waist hip ratio adjusted BMI. APC: antigen presenting cell, FC: frontal cortex, GP: globus pallidus, IT: intratelencephalic, L: layer, OPC: oligodendrocyte precursor cell, PC: posterior cortex, PT: pyramidal tract, PVM: perivascular macrophage, SMC: smooth muscle cell, SN: substantia nigra, TH: thalamus, VLMC: vascular leptomeningeal cell, VSM: vascular smooth muscle.

Supplementary Tables

Supplementary Table 1. Threshold of P-value and proportional significance for conditional analysis

Scenario (priority)	Cell type A	Cell type B	Cell type A state	Cell type B state	Description
1	$PS_{A,B} \geq 0.8$	$PS_{B,A} \geq 0.8$	indep	indep	The association of cell type A and B are independent.
2	$p_{A,B} \geq 0.05$	$p_{B,A} \geq 0.05$	joint	joint-drop	The association of cell type A and B are depending each other, and the model cannot distinguish association of two cell types. In this case, cell type A is retained and B is dropped as cell type A has more significant marginal P-value, but it does not mean association of cell type A is true and B is not.
3	$PS_{A,B} < 0.2$	$PS_{B,A} < 0.2$	joint	joint-drop	Similar to the scenario 2, but the association of cell type A and B are not completely explained by each other. In this case, only cell type A is retained as the significance of cell type B drop to less than 20% of the marginal association. The output (state of cell types) is exactly the same as scenario 2, however there might be still some signals specific to each cell type A and B.
4	$PS_{A,B} \geq 0.5$	$p_{B,A} \geq 0.05$	main	drop	The association of cell type B is completely depending on the association of cell type A. Only cell type A is retained.
5	$PS_{A,B} \geq 0.8$	$PS_{B,A} < 0.2$	main	partial-drop	The association of cell type B is mostly depending on the association of cell type A but cell type A cannot completely explain the association of cell type B. In this case, only cell type A is retained as the significance of cell type B drop to less than 20% of the marginal association, however there are a small number of signals remained (since P-value is still less than 0.05).
6	$PS_{A,B} \geq 0.5$	$PS_{B,A} \geq 0.5$	partial-joint	partial-joint	The association of cell type A and B are only partially explained by each other but majority of signals are coming from the independent associations. Both cell type A and B are retained.
7	$PS_{A,B} \geq 0.2$	$PS_{B,A} \geq 0.2$	partial-joint	partial-joint-drop	Similar to scenario 6 but larger proportion of the signals are explained by each other. In this case, only cell type A is retained as cell type B remain less than 20% of marginal significance, however there might still be specific underlying signal for cell type B.
8	$PS_{A,B} \geq 0.5$	$PS_{B,A} < 0.2$	partial-joint	joint-drop	The association of cell type B is mostly explained by cell type A but there is a part of association dependent on both cell types. In this case, only cell type A is retained.

Supplementary Table 2. GWAS summary statistics for 26 traits

Disease/Trait	Abbreviation	Ref (PMID)	N	Reference panel	Link
**Cardiovascular**					
Coronary artery disease	CAD	Nikpay et al. 2015[1]	184305	1000G	http://www.cardiogramplusc4d.org/media/cardiogramplusc4d-consortium/data-downloads/cad.additive.Oct2015.pub.zip
Diastolic blood pressure	DBP	Watanabe et al. 2018[2]	361411	UKB2	http://atlas.ctglab.nl/ukb2_sumstats/f.4079.0.0_res.EUR.sumstats.MACfilt.txt.gz
High blood pressure	HBP	Watanabe et al. 2018[2]	385699	UKB2	http://atlas.ctglab.nl/ukb2_sumstats/6150_4_logistic.EUR.sumstats.MACfilt.txt.gz
Pulse pressure	PR	Watanabe et al. 2018[2]	361411	UKB2	http://atlas.ctglab.nl/ukb2_sumstats/f.102.0.0_res.EUR.sumstats.MACfilt.txt.gz
Systolic blood pressure	SBP	Watanabe et al. 2018[2]	361402	UKB2	http://atlas.ctglab.nl/ukb2_sumstats/f.4080.0.0_res.EUR.sumstats.MACfilt.txt.gz
**Immunological**					
Inflammatory bowel disease	IBD	Lange et al. 2017[3]	59957	1000G	ftp://ftp.sanger.ac.uk/pub/project/humgen/summary_statistics/human/2016-11-07/ibd_build37_59957_20161107.txt.gz
Multiple Sclerosis	MS	Sawcer et al. 2011[4]	27148	1000G	https://www.immunobase.org/downloads/protected_data/GWAS_Data/hg19_gwas_ms_imsgc_4_19_1.tab.gz
Rheumatoid arthritis	RA	Okada et al. 2014[5]	103638	1000G	https://grasp.nhlbi.nih.gov/downloads/ResultsOctober2016/Okada/RA_GWASmeta_TransEthnic_v2.txt.gz
Systemic lupus erythematosus	SLE	Bentham et al. 2015[6]	14267	1000G	https://www.immunobase.org/downloads/protected_data/GWAS_Data/hg19_gwas_sle_bentham_4_20_0.tab.gz
Type 1 diabetes	T1D	Bradfield et al. 2011[7]	26890	1000G	https://www.immunobase.org/downloads/protected_data/GWAS_Data/hg19_gwas_t1d_bradfield_4_19_1.tab.gz
**Metabolic**					
Body fat percentage	BF	Lu et al. 2016[8]	100716	1000G	http://walker05.u.hpc.mssm.edu/body_fat_percentage_GWAS_PLUS_MC_ALL_ancestry_se_Sex_combined_for_locus_zoom_plot.TBL.txt
Body mass index	BMI	Pulit et al. 2018[9]	806834	UKB2	https://zenodo.org/record/1251813/files/bmi.giant-ukbb.meta-analysis.combined.23May2018.txt.gz?download=1

Chronic kidney disease	CKD	Pattaro et al. 2016[10]	117165	1000G	https://fox.nhlbi.nih.gov/CKDGen/formatted_round3meta_CKD_overall_IV_2GC_b36_MAFget005_Nget50_20120725_b37.csv.gz
Obesity (class1)	OB	Berndt et al. 2013[11]	98697	1000G	http://portals.broadinstitute.org/collaboration/giant/images/4/42/GIANT_OBESITY_CLASS1_Stage1_Berndt2013_publicrelease_HapMapCeuFreq.txt.gz
Waist hip ratio (adj. BMI)	WHR	Pulit et al. 2018[9]	694649	UKB2	https://zenodo.org/record/1251813/files/whradjbmi.giant-ukbb.meta-analysis.combined.23May2018.txt.gz?download=1
**Cognitive**					
Educational attainment	EA	Lee et al. 2018[12]	766345	UKB2	https://www.dropbox.com/s/ho58e9jmytmpaf8/GWAS_EA_excl23andMe.txt?dl=0
Intelligence	IQ	Savage et al. 2018[13]	269441	UKB2	https://ctg.cncr.nl/software/summary_statistics
**Neurological**					
Alzheimer disease	AD	Jansen et al. 2019[14]	74046	UKB2	https://ctg.cncr.nl/software/summary_statistics
Epilepsy	EPL	Anney et al. 2014[15]	34853	1000G	http://www.epigad.org/gwas_ilae2014/ILAE_All_Epi_11.8.15.txt.gz
Insomnia	ISM	Jansen et al. 2019[16]	386533	UKB2	https://ctg.cncr.nl/software/summary_statistics
Intracranial Volume	IV	Adams et al. 2016[17]	26577	1000G	http://enigma.ini.usc.edu/research/download-enigma-gwas-results/
**Psychiatric**					
Attention deficit hyperactivity disorder	ADHD	Demontis et al. 2018[18]	53293	1000G	https://www.med.unc.edu/pgc/results-and-downloads/downloads
Major depressive disorder	MDD	Wray et al. 2018[19]	480359	UKB1	https://www.med.unc.edu/pgc/results-and-downloads/downloads
Neuroticism	NEU	Nagel et al. 2018[20]	389206	UKB2	https://ctg.cncr.nl/software/summary_statistics
Schizophrenia	SCZ	Pardinas et al. 2018[21]	105318	1000G	http://walters.psycm.cf.ac.uk/clozuk_pgc2.meta.sumstats.txt.gz
Subjective well being	SWB	Okbay et al. 2016[22]	298420	1000G	http://ssgac.org/documents/SWB_Full.txt.gz

Supplementary Note

1. Effects of average expression across cell types

In our model, we added the average expression across cell types in a dataset as a covariate:

$$Z = \beta_0 + E_c\beta_E + A\beta_A + B\beta_B + \varepsilon \qquad (1)$$

We compared this model with a model without conditioning on average expression across cell types:

$$Z = \beta_0 + E_c\beta_E + B\beta_B + \varepsilon \qquad (2)$$

In these equations, Z is a gene-based Z-score converted from the gene-based P-value, B is a matrix of several technical confounders, E_c is the log transformed gene expression value of a testing cell type c, and A is the average log expression across cell types in a dataset. We used three traits (CAD, IBD and SCZ) and the Tabula Muris FACS dataset to compare these two models with and without conditioning on A. **Supplementary Figure 2** clearly shows that, when the model does not correct for A, a larger proportion of cell types resulted in stronger association P-values compared to the model that does correct for A. This means that the genetic associations have a general positive relationship with genes that are highly expressed in any cell type. Yet higher expression in any cell type does not necessary imply cell specificity (e.g. genes can be highly expressed in all of cell types compared to other genes). Therefore, correcting for the average expression across cell types is important when identifying cell type specificity, and drawing conclusion on the association of a specific cell type with a trait.

2. Comparison with the model from the study of Skene *et al.*

In the study of Skene *et al.*, the expression value per gene (i.e. UMI count or CPM) was converted into a specificity score (S score) as $S_c = D_c / \sum_{i \in C} D_i$, where D_c is the average expression (without log transformation) of cell type c and C = {cell type 1, cell type 2, …}[23]. They further grouped the S score into 40 bins per cell type (**Methods**) and then performed the gene-property analysis with MAGMA, using the regression model:

$$Z = \beta_0 + S_{c,bin}\beta_S + B\beta_B + \varepsilon \qquad (3)$$

Here, $S_{c,bin}$ is the binned S score and a one-sided test $\beta_S>0$ is performed.

By construction, the S score contains a correction for average expression across tissues for each gene, since for each gene, that average is $D_{\text{AVG}} = \sum_{i \in C} D_i / N_c$ (with N_c the number of cell types) and so S_c is proportional to D_c / D_{AVG}. Because the binning of the values is only dependent on their order, and log transformation is order-preserving, it is therefore equivalent

to perform the binning on a modified S score $L_c = \log D_c / D_{\text{AVG}} = \log D_c - \log D_{\text{AVG}} = E_c - \log D_{\text{AVG}}$.

One major problem with this approach is that it makes a strong assumption about the relation the average expression has with genetic association with the phenotype. This becomes apparent if we consider the same model without binning:

$$Z = \beta_0 + L_c\beta_S + B\beta_B + \varepsilon = \beta_0 + E_c\beta_S - (\log D_{\text{AVG}})\beta_S + B\beta_B + \varepsilon \quad (4)$$

The $\log D_{\text{AVG}}$ variable (log of the average expression across cell types) differs from the A variable (average of the log expression across cell types) used in our model, but they are conceptually similar in that they reflect a general expression level of genes across the cell types. However, where in our model this general expression level has a separate parameter β_A, the effect of the general expression under this approach is constrained to be equal to $-\beta_S$, the effect of E_c. If this constraint is not true for a particular phenotype and cell type, this is therefore very likely to not fully correct for the effect of the general expression level. As a result, β_S and the corresponding P-value will not truly reflect a cell type specific effect.

To study the effect of adequately or inadequately correcting for A and binning the expression values, we compared the following six different models using Tabula Muris FACS dataset and tested for three GWAS summary statistics (CAD, IBD and SCZ).

Model 1: log transformed expression value (E_c) without correcting for the average log expression across cell types

$$Z = \beta_0 + E_c\beta_E + B\beta_B + \varepsilon \quad (5)$$

Model 2: same as Model 1, but correcting for the average log expression across cell types (main model used in this study)

$$Z = \beta_0 + E_c\beta_E + A\beta_A + B\beta_B + \varepsilon \quad (6)$$

Model 3: binned log transformed expression value ($E_{c,bin}$) without correcting for the average log expression across cell types

$$Z = \beta_0 + E_{c,bin}\beta_E + B\beta_B + \varepsilon \quad (7)$$

Model 4: same as Model 3 with correcting for the binned average log expression across cell types (A_{bin})

$$Z = \beta_0 + E_{c,bin}\beta_E + A_{bin}\beta_A + B\beta_B + \varepsilon \quad (8)$$

Model 5: S score (S_c)

$$Z = \beta_0 + S_c\beta_S + B\beta_B + \varepsilon \quad (9)$$

Model 6: binned S score ($S_{c,bin}$; model used in the study of Skene *et al.*[23]); this is equivalent to using the binned modified S score $L_{c,bin}$

$$Z = \beta_0 + S_{c,bin}\beta_S + B\beta_B + \varepsilon = \beta_0 + L_{c,bin}\beta_S + B\beta_B + \varepsilon \quad (10)$$

In the previous section (**Supplementary Note 1**), we showed that the model without conditioning on the general expression (Model 1) leads to an inflation of the association statistics compare to the model with conditioning on the general expression (Model 2; **Supplementary Figure 3**). The binning of the expression value resulted in a slight inflation of association statistics by comparing Model 1 and 3 (**Supplementary Figure 3**). Similar inflation was seen by binning the S score when comparing Model 5 and 6. However, by conditioning on the average of the binned expression value (Model 4), the association statistics were very similar to the model without binning (Model 2; **Supplementary Figure 3**).

We also show that the number of bins does not strongly affect the p-values (**Supplementary Figure 4**). By comparing Model 2 to Model 4, which differ only in respect to binning but results for these models were very similar. Thus, the binning itself does not lead to an inflation but will do so in the context of insufficiently (or not at all) correcting for average expression, because the binned scores are not independent from the average expression values.

Taken together, these analyses indicate that correcting for the general expression level across cell types independently from cell specific expression has a similar effect on the results regardless of the binning of expression value. When using such a correction the cell type associations will tend to be weaker, suggesting that inflated associations found in the uncorrected models are in part not cell type specific.

Consequently, the reason why the binned S score analysis in Skene *et al.* showed generally stronger associations with many of the traits is that there can be a strong positive correlation between $S_{c,bin}$ and A depending on cell types. Because neither A nor another variable reflecting the general expression level was not independently corrected in the model, any effect of general expression will result in an inflation the association of $S_{c,bin}$.

For example, $S_{c,bin}$ of B-cell from Lung has a relatively strong positive correlation with A (r=0.52; **Supplementary Figure 5a**), and was significantly associated with CAD by Model 6 but not by Model 2. Similarly, $S_{c,bin}$ of granulocyte from Marrow is positively correlated with A (r=0.44; **Supplementary Figure 5b**), and was significantly associated with IBD by Model 6 but not by Model 2. These two significant enrichments in Model 6 are due to genes which are highly expressed across cell types as those associations dropped when correcting for A.

In contrast, neurons showed low correlation with *A* (r=0.04; **Supplementary Figure 5c**) which was significantly associated with SCZ by both Model 2 and Model 6, but not associated with CAD and IBD in either model. This is therefore likely a true signal independent from the general expression of genes across cell types.

We showed that cell type specific expression values which have a strong correlation with the general expression level across cell types can exhibit a significant association with the trait, even if there is no effect specific to expression in that cell type. Therefore, independently correcting for the general expression level is crucial to identify true cell specificity associations, and to allow drawing conclusions on cell type specific trait associations. Due to the way the S score used by Skene *et al.* was defined, general expression effects were likely not fully corrected for in their analyses, and as such it is uncertain to what extent the significant associations they found truly reflect cell type specific effects.

3. Comparison with stratified LD score regression and RolyPoly

We compared the MAGMA regression model (Model 2 in the previous section) with previously proposed methods, LD score regression (LDSC)[24] and RolyPoly[25]. In this section, we used 119 cell types from Tabula Muris FACS datasets with GWAS summary statistics of CAD, IBD and SCZ.

The cell type specificity analysis with LDSC is based on the stratified LDSC model for a certain genomic annotation[24]. By regressing chi-square association statistics of SNPs on an LD score of SNPs in an annotation to be tested, the regression coefficient reflects the importance of the annotation. To test cell type specificity, LDSC evaluates whether the regression coefficient is greater than zero[24]. In the case of cell type specificity analysis, the genomic annotation is defined by cell type specific genes with extended windows[24]. We defined cell type specific genes for each cell type by taking the top 10% of genes with the highest S score as previously applied[23]. We also created another set of cell type specific genes by takin the top 10% of genes with the highest residuals of gene expression after regressing out the average expression across cell types which is more comparable with MAGMA regression model (**Methods**). We performed LDSC for each cell type twice (with different definitions of cell type specific genes) and each result are compared with MAGMA results separately. We observed that the top significantly associated cell types tend to be similar when using MAGMA and LDSC, for IBD and SCZ (**Supplementary Figure 7**). We

do note that the MAGMA analysis resulted in a larger number of significant associations compared to LDSC (**Supplementary Figure 7**).

RolyPoly is also designed to identify trait associated cell types using gene expression profiles and GWAS summary statistics. RolyPoly implements a polygenic model and estimates parameters which capture the influence of each gene on the variance of GWAS effect sizes for each cell type, given gene expression matrix[25]. The software then performs bootstrap to estimate standard errors used to compute a test statistic. Due to the highly intensive computation for bootstrapping, we limited the analysis (only in this section) to chromosomes 10-22. The results showed that RolyPoly often identified a much lower number of significant associations compared to the MAGMA regression model (**Supplementary Figure 8**).

4. FDR versus Bonferroni multiple testing correction

The choice of multiple test correction can potentially result in different conclusions of cell type associations. In this study we employed Bonferroni correction for all tested cell types across datasets to minimize false positive findings. However, one could argue that since there might be the same cell types in the different datasets, applying a Bonferroni correction may be over correcting. Indeed FDR correction has been used in previous studies[24]. Although the number of significant cell types retained from step 1 in our workflow can notably change by using FDR instead of Bonferroni correction, retained cell types after per dataset conditional analysis in step 2 are likely to remain similar as the majority of the P-value of cell types that are below FDR threshold but above the Bonferroni threshold will increase (i.e. become less significant) after conditioning on the most significant cell type in the same dataset (**Supplementary Figure 10**). However, this does not apply to datasets with cell types that only reach significance after FDR correction but not after Bonferroni correction. Therefore, by using FDR, there will likely be a larger number of cell types retained from a larger number of datasets compared to Bonferroni correction. In this study, we used Bonferroni correction to minimize false positive findings.

5. Detailed interpretation of multi-conditional analyses

Coronary artery disease (CAD)

By using 43 scRNA-seq datasets, 68 cell types from 14 datasets reached significance after Bonferroni correction (Step 1, **Supplementary Data 5.1**). After conditional analyses per dataset (step 2), 16 cell types (from 14 datasets) were retained; 13 endothelial cells and 2

astrocytes and muscle cells (**Supplementary Data 5.2-5.3**). By conditioning on the cell type with the most significant marginal P-value with CAD (i.e. endothelial cell from Aleen_mm_VISp), 10 endothelial cells remained less than 50% of CD marginal significance, indicating their associations are either mainly derived or jointly explained by the most significant association (**Fig. 5a** and **Supplementary Data 5.4**). The endothelial cell from Allen_mm_ALM dataset was not able to complete the conditional analyses due to collinearity, while pancreatic endothelial cell from GSE84133_hs dataset remained more than 50% of CD marginal significant after conditioning (**Fig. 5a**).
Associations of astrocytes from brain-specific datasets and muscle cell from multi-tissue datasets were independent from endothelial cells (both remained >60% of CD marginal significance; **Fig. 5a** and **Supplementary Data 5.4**).

Inflammatory Bowel Disease (IBD)
Involvement of immune system in IBD is well established[26,27] and tissue specificity analysis with GTEx shows significant associations with whole blood and tissues enriched by immune cells (**Supplementary Data 4**). Using scRNA-seq datasets, IBD showed significant associations with 104 cell types from 22 datasets (**Supplementary Data 5.21**). After the second step, 25 cell types were retained (**Supplementary Data 5.22-23**). Eighyrrn of them were microglia, and the remaining cells were several immune cells including macrophage, T cell, B cell, blood cell and endothelial cell. By conditioning on the most significant association (microglia from Allen_hs_MTG), the associations of 19 other microglia and macrophage decreased to less than 50% of CD marginal significance (**Supplementary Figure 12i**). While several immune cell types and endothelial cell retained most of the CD marginal significance (**Supplementary Figure 12i)**. Of which, tracheal blood cell from TMF showed the next most significant marginal P-value. The association of this cell type was the main driver for liver T-cell from Mouse Cell Atlas and jointly associated with mammary macrophage from Tabula Muris droplet (**Supplementary Figure 12i**). In contrast, marrow B-cell and granulocyte from Tabula Muris FACS can only be partially explained by trachea blood cell (**Supplementary Figure 12i**). In addition, endothelial cell from DropViz showed independent associations with most of the other immune cells (**Supplementary Figure 12i**). These results indicate that three are independent signals specific to microglia, blood cells, B-cells, granulocyte and endothelial cells. Involvement of B-cells and granulocyte in IBD have

been widely reported [28,29] and, dysfunction of endothelial cells as well as interaction with immune cells have been reported to be one of the ethological factors of IBD[30,31].

Schizophrenia (SCZ)

We identified significant associations of SCZ with 187 cell types from 20 datasets including multiple neuronal cell types (**Fig. 5c** and **Supplementary Data 5.53**). The most significant marginal association was seen in neurons from TMF. There were four non-brain related cell types (pancreatic endocrine cells from TMF) significantly associated with SCZ, however these associations were completely dependent on association of neuron from the same dataset (**Supplementary Data 5.54**). After within dataset conditional analyses (step 2), 25 cell types were retained (**Supplementary Data 5.54-5.55**). By conditioning on the cell type with the most significant marginal P-value (i.e. neurons from TMF) across datasets, associations of 12 neuronal cell types from different datasets decreased less than 50% of CD marginal significance (**Fig. 5b** and **Supplementary Data 5.56**). As TMF is multi-tissue dataset while most of other datasets are brain-specific, the strong association might be due to the difference of the cell types available in the dataset (discussed in the main text). In other words, the results indicate a strong association of SCZ with broadly defined neurons rather than specific sub-types of the neuronal cells. Nine other cell types remained more than 50% of CD marginal significance (**Fig. 6b** and **Supplementary Data 5.56**). Two of them were GABAergic neurons and lateral neuroblast from human and mouse embryo brain samples (GSE76381) suggesting there are signals specific to embryonic brain cells associated with SCZ independent from neurons of adult samples. Embryonic development of brain has been associated with risk of SCZ [32,33]. We also observed independent cluster of 5 excitatory neurons including Layer 4-5. A specific subtype of neuron from DropViz dataset also showed independent association from other neurons. The remaining cell is HBINH2 (sub-cluster of inhibitory neurons from hindbrain) from MBA indicating independent signals of inhibitory neurons from (excitatory) neurons.

Supplementary References

1. Nikpay, M. *et al.* A comprehensive 1000 Genomes-based genome-wide association meta-analysis of coronary artery disease. *Nat. Genet.* **47,** 1121–1130 (2015).
2. Watanabe, K. *et al.* A global view of pleiotropy and genetic architecture in complex traits. Preprint at https://doi.org/10.1101/500090 (2018).
3. De Lange, K. M. *et al.* Genome-wide association study implicates immune activation of multiple integrin genes in inflammatory bowel disease. *Nat. Genet.* **49,** 256–261 (2017).
4. Sawcer, S. *et al.* Genetic risk and a primary role for cell-mediated immune mechanisms in multiple sclerosis. *Nature* **476,** 214–219 (2011).
5. Okada, Y. *et al.* Genetics of rheumatoid arthritis contributes to biology and drug discovery. *Nature* **506,** 376–81 (2014).
6. Bentham, J. *et al.* Genetic association analyses implicate aberrant regulation of innate and adaptive immunity genes in the pathogenesis of systemic lupus erythematosus. *Nat. Genet.* **47,** 1457–1464 (2015).
7. Bradfield, J. P. *et al.* A genome-wide meta-analysis of six type 1 diabetes cohorts identifies multiple associated loci. *PLoS Genet.* **7,** e1002293 (2011).
8. Lu, Y. *et al.* New loci for body fat percentage reveal link between adiposity and cardiometabolic disease risk. *Nat. Commun.* **7,** 10495 (2016).
9. Pulit, S. L. *et al.* Meta-analysis of genome-wide association studies for body fat distribution in 694,649 individuals of European ancestry. *Hum. Mol. Genet.* **28,** 166–174 (2018).
10. Pattaro, C. *et al.* Genetic associations at 53 loci highlight cell types and biological pathways relevant for kidney function. *Nat. Commun.* **7,** 10023 (2016).
11. Berndt, S. I. *et al.* Genome-wide meta-analysis identifies 11 new loci for anthropometric traits and provides insights into genetic architecture. *Nat. Genet.* **45,** 501–512 (2013).
12. Lee, J. J. *et al.* Gene discovery and polygenic prediction from a genome-wide association study of educational attainment in 1.1 million individuals. *Nat. Genet.* **50,** 1112–1121 (2018).
13. Savage, J. E. *et al.* GWAS meta-analysis (N=279,930) identifies new genes and functional links to intelligence. *Nat. Genet.* **50,** 912–919 (2018).
14. Jansen, I. E. *et al.* Genome-wide meta-analysis identifies new loci and functional

pathways influencing Alzheimer's disease risk. *Nat. Genet.* **51,** 404–413 (2019).

15. Anney, R. J. L. *et al.* Genetic determinants of common epilepsies: A meta-analysis of genome-wide association studies. *Lancet Neurol.* **13,** 893–903 (2014).
16. Jansen, P. R. *et al.* Genome-wide analysis of insomnia in 1,331,010 individuals identifies new risk loci and functional pathways. *Nat. Genet.* **51,** 394–403 (2019).
17. Adams, H. H. H. *et al.* Novel genetic loci underlying human intracranial volume identified through genome-wide association. *Nat. Neurosci.* **19,** 1569–1582 (2016).
18. Demontis, D. *et al.* Discovery of the first genome-wide significant risk loci for attention deficit/hyperactivity disorder. *Nat. Genet.* **51,** 63–57 (2018).
19. Wray, N. R. *et al.* Genome-wide association analyses identify 44 risk variants and refine the genetic architecture of major depression. *Nat. Genet.* **50,** 668–681 (2018).
20. Nagel, M. *et al.* Meta-analysis of genome-wide association studies for neuroticism in 449 , 484 individuals identifies novel genetic loci and pathways. *Nat. Genet.* **50,** 920–927 (2018).
21. Pardiñas, A. F. *et al.* Common schizophrenia alleles are enriched in mutation-intolerant genes and in regions under strong background selection. *Nat. Genet.* **50,** 381–389 (2018).
22. Okbay, A. *et al.* Genetic variants associated with subjective well-being, depressive symptoms, and neuroticism identified through genome-wide analyses. *Nat. Genet.* **48,** 624–633 (2016).
23. Skene, N. G. *et al.* Genetic identification of brain cell types underlying schizophrenia. *Nat. Genet.* **50,** 825–833 (2018).
24. Finucane, H. K. *et al.* Heritability enrichment of specifically expressed genes identifies disease-relevant tissues and cell types. *Nat. Genet.* **50,** 621–629 (2018).
25. Calderon, D. *et al.* Inferring relevant cell types for complex traits by using single-cell gene expression. *Am. J. Hum. Genet.* **101,** 686–699 (2017).
26. De Mattos, B. R. R. *et al.* Inflammatory bowel disease: An overview of immune mechanisms and biological treatments. *Mediators Inflamm.* **2015,** 493012 (2015).
27. Lee, S. H., Kwon, J. eun & Cho, M.-L. Immunological pathogenesis of inflammatory bowel disease. *Intest. Res.* **16,** 26–42 (2018).
28. Levine, A. P. & Segal, A. W. What Is wrong with granulocytes in inflammatory bowel diseases. *Dig. Dis.* **31,** 321–327 (2013).
29. Silva, F. A. R., Rodrigues, B. L., Ayrizono, M. D. L. S. & Leal, R. F. The

immunological basis of inflammatory bowel disease. *Gastroenterol. Res. Pract.* **2016,** 2097274 (2016).

30. Danese, S. & Fiocchi, C. Endothelial cell-immune cell interaction in IBD. *Dig. Dis.* **34,** 43–50 (2016).
31. Gravina, A. G. *et al.* Vascular endothelial dysfunction in inflammatory bowel diseases: Pharmacological and nonpharmacological targets. *Oxid. Med. Cell. Longev.* **2018,** 2568569 (2018).
32. Selemon, L. D. & Zecevic, N. Schizophrenia: A tale of two critical periods for prefrontal cortical development. *Transl. Psychiatry* **5,** e623 (2015).
33. Stachowiak, E. K. *et al.* Cerebral organoids reveal early cortical maldevelopment in schizophrenia-computational anatomy and genomics, role of FGFR1. *Transl. Psychiatry* **7,** 6 (2017).
