# Supplementary material for: Genetic mapping of cell type specificity for complex traits
Source: Nat Commun. 2019 Jul 19;10:3222. doi: 10.1038/s41467-019-11181-1 (PMC6642112; doi:10.1038/s41467-019-11181-1)
Supplement: Supplementary file 4 — Description of Additional Supplementary Files [file 41467_2019_11181_MOESM4_ESM.pdf]

Description of Additional Supplementary Files

Supplementary Data 1.
scRNA-seq datasets
Supplementary Data 2.
Details of scRNA-seq datasets pre-process
Supplementary Data 3.
Cell type description and tSNE coordinates
Supplementary Data 4.
Tissue specificity analyses with GTEx v7 53 tissues.
Supplementary Data 5.
Full results of cell type specificity analyses for 26 traits.
